# Self-Reported Health Outcomes in Metabolic Health YouTube Comments: Cross-Sectional Study and Rule-Based Natural Language Processing Framework Development and Validation

**DOI:** 10.2196/94855

**Published:** 2026-05-26

**Authors:** Ricardo Ribeiro, Aneesh Zutshi

**Affiliations:** 1 Department of Mechanical and Industrial Engineering NOVA School of Science and Technology Universidade Nova de Lisboa Caparica, Lisbon Portugal

**Keywords:** healthcasting, therapeutic carbohydrate restriction, user-generated content, health outcomes, natural language processing, ontology engineering, precision-optimized extraction, YouTube, self-reported outcomes, metabolic health, digital health

## Abstract

**Background:**

YouTube is increasingly used for healthcasting, the sharing of evidence-based dietary and lifestyle interventions by domain experts. In the metabolic health domain, channels focused on therapeutic carbohydrate restriction have accumulated audiences of millions. A distinctive feature is the comment section, where viewers share first-person accounts of health changes, constituting a unique source of real-world outcome data at scale. However, extracting structured health information from unstructured comments presents computational challenges.

**Objective:**

This observational, cross-sectional study aims to develop and validate a precision-optimized computational framework for extracting self-reported health outcomes from healthcasting YouTube comments and to characterize the prevalence, distribution across health aspects, and channel-level variation of reported outcomes across a large-scale metabolic health corpus.

**Methods:**

This study analyzed 43,111 unique YouTube comments from 110 videos across 11 therapeutic carbohydrate restriction-focused healthcasting channels (37,458 unique authors; data span November 2013 to January 2026; collected via YouTube data application programming interface version 3). The methodology comprised 3 construction phases and 5 validation studies. The construction phases were (1) exploratory corpus characterization, (2) iterative development of a 35-aspect hierarchical health outcome ontology, and (3) precision-optimized rule-based classification, validated through precision validation (stratified sample of n=500), recall estimation (n=510), external validation on 5 held-out channels (n=12,653 comments), large language model–assisted interrater reliability assessment, and transformer baseline comparison against Bidirectional Encoder Representations from Transformers (BERT) and Robustly Optimized BERT Pretraining Approach (ROBERTa) classifiers. A supplementary aspect–based sentiment analysis contextualized the positive-only design.

**Results:**

The framework identified 1790 positive health outcome reports (1790/43,111, 4.15% prevalence), achieving 97.6% (488/500) precision (95% CI 95.7%-98.6%) and estimated 56.2% recall (95% CI 43.4%-67.9%). The reports described 6674 positive outcomes, distributed across 35 health aspects and 18 named disease conditions extending beyond weight loss: pain and inflammation reduction (1137/6674, 17%), type 2 diabetes improvement (977/6674, 14.6%), skin health (784/6674, 11.8%), and psychological well-being (731/6674, 11%). Over half (3355/6674, 50.3%) spanned multiple research objectives. Significant channel-level variation was observed (χ²_10_=927.5; *P*<.001), with positive outcome rates ranging from 1.32% to 10.40% (odds ratio 8.68, 95% CI 7.10-10.61). Transformer baselines achieved higher recall but lower precision, confirming their advantage for high-confidence corpus generation. A supplementary aspect-based sentiment analysis indicated a positive-to-negative ratio of approximately 4.6:1 (n=1003), with negative experiences (59/495, 11.9%) predominantly involving gastrointestinal and cardiovascular concerns.

**Conclusions:**

This study presents, to our knowledge, the first validated, rule-based framework for extracting self-reported metabolic health outcomes from healthcasting YouTube comments at corpus scale. Unlike existing recall-oriented social media health classifiers, the precision-optimized design achieves the confidence threshold required for outcomes research without manual review. These findings demonstrate that expert-led health content comment sections constitute a scalable, complementary data source for monitoring real-world engagement with dietary interventions, with implications for public health surveillance, platform design, and health communication research.

## Introduction

### Background and Motivation

Ongoing advances in metabolic health research have identified insulin resistance and excessive glycemic variability as principal contributors to chronic systemic inflammation and metabolic stress [1]. Therapeutic carbohydrate restriction (TCR), encompassing ketogenic, low-carbohydrate, carnivore, and intermittent fasting approaches, reduces dietary carbohydrate to shift metabolic fuel use toward fatty acid oxidation and ketone body production [2]. TCR-based interventions have demonstrated clinically significant improvements in glycemic control, body composition, and cardiometabolic risk markers across multiple randomized controlled trials and systematic reviews [3-8], with a recent meta-analysis of 30 randomized controlled trials (3806 adults) reporting significant reductions in metabolic syndrome indicators [9]. Research has extended into neurological applications [10,11] and metabolic psychiatry, where pilot clinical data suggest that ketogenic interventions may improve psychiatric symptom severity in bipolar disorder and schizophrenia [12-16].

Despite this growing evidence base, several structural challenges constrain the conduct of large-scale clinical trials on dietary interventions. Dietary trials are inherently difficult to blind, compliance monitoring is resource-intensive, and long-term adherence remains a persistent methodological challenge [10,17]. Critically, because TCR interventions involve dietary and lifestyle modification rather than pharmaceutical compounds, there is no direct commercial entity positioned to sponsor large-scale efficacy trials comparable to those conducted for pharmacological interventions [6]. This funding asymmetry does not reflect a lack of scientific interest or clinical signal, but rather the structural economics of nutrition research [6].

Concurrently, a substantial population is adopting TCR-based dietary approaches outside formal clinical settings [18,19], informed by credentialled expert content disseminated through YouTube. Over the past decade, a distinct health communication phenomenon has emerged [20]: expert-led channels in which physicians, researchers, and clinicians share evidence-based dietary interventions directly with lay audiences at scale [21]. We term this phenomenon healthcasting: the systematic delivery of health education through video platforms by domain experts, bypassing traditional clinical and media gatekeeping structures [22]. We adopt this compound term to distinguish the specific phenomenon of expert-led health content creation with bidirectional outcome reporting from broader categories such as health podcasting or medical influencing. In the metabolic health domain, healthcasting channels focused on TCR have accumulated audiences in the millions, with comment engagement growing from a few hundred interactions per year in 2017 to more than 73,000 comments in 2024 across the 11 channels examined in this study. Because TCR interventions are dietary rather than pharmaceutical, they are uniquely amenable to self-directed implementation [18,19], making this domain one of the most developed examples of research-to-audience healthcasting. The purpose of this paper is to extract and analyze user-reported health outcomes within this specific approach, not to compare TCR with alternative interventions.

Beneath these videos, many viewers post comments reporting personal health changes, frequently including temporal markers suggesting longitudinal self-monitoring (eg, “after 5 weeks... my fatty liver is reversed”) [23]. While each comment is classified independently, the prevalence of temporal language provides indirect evidence that commenters report outcomes observed over weeks to months of dietary change. These comments constitute unsolicited, real-world, naturalistic health outcome data not available in any clinical registry, representing self-reported experiences of individuals who encountered expert content, acted on it, and publicly documented the results [24].

Several important caveats apply to the interpretation of self-reported health outcomes extracted from social media commentary. Such data are subject to selection bias [25] (individuals who experience positive outcomes may be more likely to comment), survivorship bias (those who discontinued may not return to report), recall bias [26], and the absence of clinical verification [24,27]. The data do not constitute clinical evidence in the conventional sense, cannot establish causal relationships between dietary interventions and health outcomes, and should not be interpreted as demonstrating clinical efficacy.

The purpose of this study is to identify which health conditions users of TCR-focused healthcasting content self-report as improved and to examine factors that may influence the distribution of these reports. The case of metabolic health and TCR was selected because it represents one of the most developed and active domains of healthcasting, with sufficient comment volume and content creator diversity to support computational analysis at scale [18,20].

### Research Gap

Health information extraction from social media [28,29] has focused predominantly on pharmacovigilance and adverse drug event detection [30-33], with the social media mining for health applications shared tasks expanding from rule-based systems to large language models (LLMs) [34]. Research has also examined Reddit mental health communities [35] and YouTube health video quality [21,36,37], and YouTube video comments on dietary topics have been examined using text mining approaches [38]. Systematic mining of YouTube comment sections for self-reported health outcomes, particularly dietary interventions, has not been addressed. This represents a gap in both health informatics methodology and our understanding of how healthcasting content translates into reported health change at the population level.

In the methodological literature, existing classification systems have been optimized predominantly for balanced *F*_1_ performance, with precision typically reported in the 80-90% range [39-41]. For applications requiring high-confidence corpus generation, where the downstream analysis depends on the validity of every included observation, this precision level is insufficient. A system that incorrectly classifies 1 in 10 or 1 in 5 comments as positive health outcomes would introduce systematic noise into any analysis of outcome distributions, disease-specific prevalence rates, or channel-level variation. The gap this study addresses is therefore twofold: the absence of a domain-specific framework for extracting self-reported health outcomes from healthcasting content, and the absence of a precision-optimized extraction methodology explicitly designed to generate validated corpora for downstream health outcomes research.

### Research Questions

This study aims to address the following research questions (RQs):

- RQ1: what is the prevalence of self-reported positive health outcomes in YouTube comments on metabolic health content?
- RQ2: what types of health outcomes are most frequently reported, and how are they distributed across subjective, objective, and disease-specific categories?
- RQ3: does positive outcome reporting vary significantly across content creators, and what factors may explain this variation?
- RQ4: can a precision-optimized rule-based framework achieve sufficient classification accuracy for generating validated health outcome corpora from user-generated content?

## Methods

### Overview

**Figure 1:**
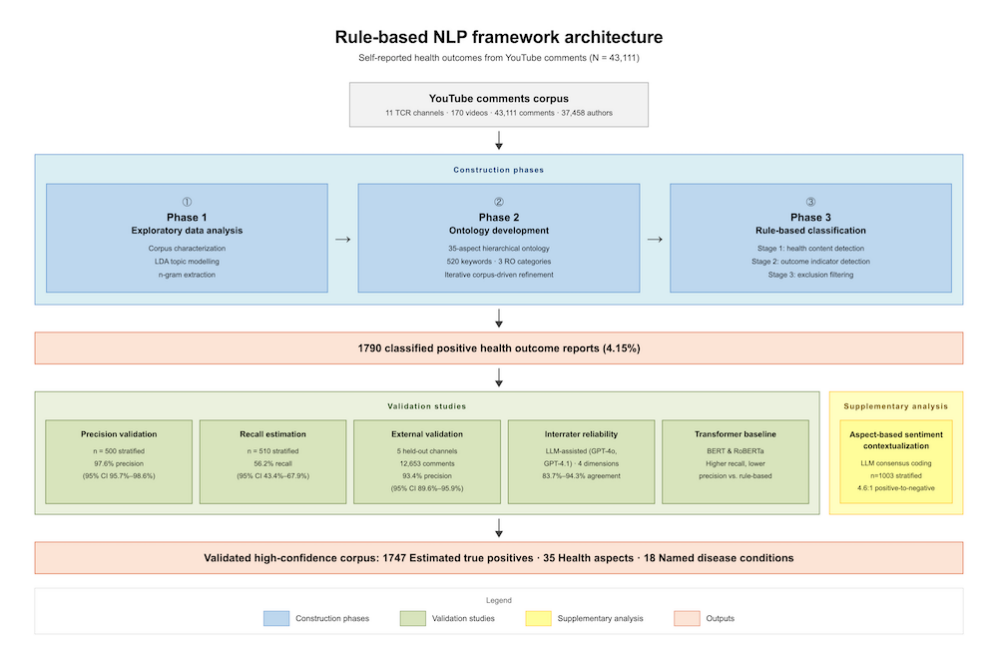
Architecture of the rule-based natural language processing framework for extracting self-reported positive health outcomes from English-language YouTube comments on metabolic health channels (N=43,111 unique comments, November 2013 to January 2026). The methodology comprises three construction phases: (1) exploratory data analysis, (2) ontology development, and (3) rule-based classification. The classifier is stress-tested through a program of Validation Studies (precision validation, recall estimation, external validation on held-out channels, interrater reliability, and transformer baseline comparison), and a supplementary analysis provides aspect-based sentiment contextualization of the positive-only design. The precision-optimized classifier uses conservative classification rules and extensive exclusion filtering. BERT: Bidirectional Encoder Representations from Transformers; LDA: latent dirichlet allocation; LLM: large language model; RO: research objective; NLP: natural language processing; RoBERTa: Robustly Optimized BERT Pretraining Approach.

The methodology comprises 3 construction phases, an integrated program of validation studies, and a supplementary contextualization analysis. The construction phases are (1) exploratory data analysis and corpus characterization, (2) ontology development through iterative pattern extraction, and (3) rule-based classification. Phase 3 is then stress-tested through five complementary validation studies: precision validation, recall estimation, external validation on held-out channels, interrater reliability assessment, and transformer baseline comparison. A supplementary aspect-based sentiment analysis (ABSA) contextualizes the primary framework’s positive-only design. Figure 1 presents the overall framework architecture.

### Research Design Overview

The framework was explicitly designed to maximize precision rather than recall. This design choice reflects the intended application: generating a high-confidence corpus of verified positive health outcomes suitable for downstream analysis. In health informatics applications, false positives (incorrectly classified outcomes) can lead to erroneous conclusions about treatment efficacy, whereas false negatives (missed outcomes) simply reduce statistical power without introducing systematic error. Following established guidance in clinical text mining [42], we prioritized precision when high-confidence annotations are required. This study follows the CREMLS (Consolidated Reporting Guidelines for Prognostic and Diagnostic Machine Learning Modeling Studies); the completed checklist is provided in Multimedia Appendix 1.

The exclusive focus on positive health outcomes reflects three considerations: positive outcomes provide the most linguistically distinctive targets, negative outcomes pose fundamentally different classification challenges (heterogeneous expressions requiring distinct approaches), and follower comment sections exhibit a structural positive bias.

The primary contributions of this work are: (1) a hierarchical ontology of 35 health aspects capturing subjective, objective, and disease-specific outcomes in TCR-focused healthcasting content; (2) a precision-optimized rule-based classification system achieving 97.6% (488/500) precision (95% CI 95.7%-98.6%); (3) a validated corpus of 1747 estimated true positive health outcome reports from 43,111 unique comments across 37,458 unique commenters; (4) comprehensive precision-recall characterization; (5) interrater reliability assessment using dual-model LLM-assisted annotation; (6) a domain-level analysis of healthcasting outcome patterns and channel-level variation; and (7) a supplementary ABSA contextualizing the positive-only extraction scope.

### Ethical Considerations

This study analyzes publicly available YouTube comments accessed through the official YouTube data application programming interface (API) version 3, in compliance with the platform’s terms of service and API use policies. No formal ethics committee review was sought, consistent with established practice in computational social media research involving publicly accessible data, where no interaction with users occurs, and no intervention is administered [43]. Several methodological and procedural safeguards were implemented to protect user privacy and ensure responsible data handling.

Data collection was limited to publicly posted comments that users understand to be visible to all internet users. No private messages, restricted content, or data requiring authentication were accessed. All processing was automated with no direct interaction with commenters, and no personally identifiable information was retained beyond publicly visible usernames used only for de-duplication. Informed consent was not required, as this study involved secondary analysis of publicly posted data with no direct interaction with or intervention upon users.

Comment excerpts presented in this study are reproduced only in truncated or paraphrased form to minimize the risk of reidentification through text search. The raw comment corpus is not included in the supplementary materials because YouTube’s terms of service restrict the redistribution of bulk API-retrieved data. The classification code, ontology, and validation protocols are made available to enable methodological reproducibility without compromising user privacy.

No compensation was provided to any participants, as this study involved secondary analysis of publicly available data and did not involve direct interaction with commenters. No images of identifiable individuals are included in this manuscript. The study was conducted in accordance with the principles of the Declaration of Helsinki applicable to observational research involving publicly available data.

### Data Collection

Comments were collected from 11 YouTube channels producing content on metabolic health and TCR. Channel selection criteria included: (1) medical or scientific credentials of content creators, (2) minimum subscriber threshold of 100,000, (3) focus on metabolic health topics, and (4) active comment sections. The YouTube Data API v3 was used to retrieve the 10 most-commented videos per channel and up to 2000 comments per video, yielding a raw corpus of 209,661 records from 110 videos. After removing duplicate records caused by API pagination (the YouTube Data API v3 returns nonunique results when paginating beyond available comments with relevance-based ordering), 43,111 unique comments were retained. Data collection was performed on January 2, 2026, capturing comments spanning November 7, 2013, to January 2, 2026. Table 1 presents corpus statistics by channel.

**Table 1:** Corpus statistics for 11 metabolic health YouTube channels included in a cross-sectional computational analysis of self-reported health outcomes (N=43,111 unique English-language comments, November 2013 to January 2026).

Channel^a^	Background^b^	Channel age (years)	Subscribers^c^ (thousands)	Views (millions)	Comments^d^	Positive outcomes	Rate (%)
KenDBerryMD	Family medicine, MD (University of Tennessee)	14.8	3660	533	3970	413	10.40
Eric Berg DC	Chiropractor, DC (Palmer College)	16.7	14,500	3350	3993	282	7.06
Eric Westman	Internal and obesity medicine, MD, PhD (University of Wisconsin), MHS (Duke)	10.1	297	36	3978	250	6.28
Jason Fung	Nephrology, MD (University of Toronto)	14.2	1410	86	3963	286	7.22
Ben Bikman	Cell biology and physiology, PhD (East Carolina University)	9.0	191	9	3476	65	1.87
Nick Norwitz	Metabolism, PhD (Oxford), MD (Harvard)	11.8	854	55	3954	82	2.07
Anthony Chaffee MD	Neurosurgery, MD (Royal College of Surgeons)	11.3	536	109	3972	104	2.62
Shawn Baker MD	Orthopedic surgery, MD (Texas Tech)	11.8	383	83	3933	52	1.32
Dr. Robert Cywes	Bariatric and pediatric surgery, MD, PhD (University of Cape Town)	6.1	307	37	3932	102	2.59
Dr. Boz	Internal medicine, MD (University of South Dakota)	13.7	1180	184	3959	86	2.17
Mark Hyman	Functional medicine, MD (University of Ottawa)	18.5	1490	144	3980	68	1.71

^a^Channels ordered by positive outcome rate.

^b^The background section lists the primary professional credentials and degree-granting institution for each content creator.

^c^Subscriber counts and total channel views were updated on March 4, 2026, to reflect current values. YouTube reports subscriber counts rounded to three significant figures.

^d^Comments data were collected via the YouTube data application programming interface, version 3, on January 2, 2026 (10 most-commented videos per channel, up to 2000 comments per video).

Video selection was maximized for content relevance and comment volume: for each channel, the 10 most-commented videos were identified using the YouTube data API. Content validity was addressed through channel credential requirements, the engagement-based selection criterion, and the classification framework’s exclusion filters. Critically, the unit of analysis is the viewer comment, not the video content itself; consequently, the classification framework’s accuracy is independent of video content quality, and no formal content quality instrument was applied to the videos. Listing for all 110 video titles, URLs, and metadata is provided in Multimedia Appendix 2. Video durations ranged from 19 seconds to 115.8 minutes (mean 23.3, SD 24.4 minutes). Short-form videos (n=13, 11.8%) were included because they met the selection criterion and contained comparable health-related discourse.

### Phase 1: Exploratory Data Analysis

Initial corpus exploration used topic modeling using latent Dirichlet allocation to identify thematic structures. N-gram analysis extracted frequently occurring bi-grams and tri-grams associated with health outcomes. Representative comments were sampled from each topic cluster to inform ontology development. This phase established the linguistic patterns characterizing health outcome reports in the corpus, distinguishing personal testimonials from general health discussions.

Corpus characterization revealed a right-skewed distribution of comment lengths (mean 32.5, SD 47 words; median 18, IQR 11-43 words), with engagement metrics confirming that most comments function as unsolicited declarations rather than conversational exchanges. Temporal analysis revealed exponential growth, rising from 3408 comments in 2019 to 73,207 in 2024.

### Phase 2: Ontology Development

The health outcome ontology was developed through an iterative, corpus-driven process in which data-derived linguistic patterns were combined with domain expert knowledge. Starting from latent Dirichlet allocation topic modeling and n-gram extraction conducted in Phase 1, candidate health concepts were identified from the most frequently occurring bi-grams and tri-grams co-occurring with outcome-indicative language (eg, “lost weight,” “blood sugar normalized,” and “pain is gone”). These candidates were then organized into a hierarchical structure of research objectives (ROs), each containing a set of thematically coherent aspects.

Three ROs were defined: RO1 captures subjective experiences (how users feel day-to-day), RO2 captures measurable biomarkers and anthropometric changes, and RO3 captures disease-level resolution (named conditions improved or reversed). The tiers are complementary and nonmutually exclusive, with 50.3% (n=3355) of 6674 reported positive outcomes spanning more than one RO.

Each aspect was defined with a unique identifier (eg, RO2.1), scope definition, inclusion keywords matched using whole-word regular expressions (case-insensitive), and exclusion patterns redirecting ambiguous matches to more specific aspects. The complete ontology comprises 35 aspects across 3 ROs, totaling 520 keywords. Table 2 presents the full structure; the complete keyword set is available in Multimedia Appendix 3.

**Table 2:** Health outcome ontology used in a rule-based natural language processing framework for extracting self-reported outcomes from English-language YouTube comments on metabolic health channels (N=43,111 unique comments, November 2013 to January 2026). The ontology comprises 35 aspects organized under three research objectives with 520 total keywords. Representative keywords are shown; the complete keyword set is available in multimedia files.

ID			Aspect name^a^		Domain; type		Scope and representative keywords
**RO1: Subjective well-being (9 aspects):** **self-reported improvements in quality of life, symptoms, and subjective health status**							
	RO1.1	Cognitive function		Subjective; neurological		Brain fog, mental clarity, memory, focus, concentration, cognitive improvement	
	RO1.2	Energy and vitality		Subjective; metabolic		Energy levels, fatigue, tiredness, lethargy, stamina, vitality, no longer tired	
	RO1.3	Psychological well-being		Subjective; mental health		Anxiety, depression, mood, stress, mental health, happiness, calm, irritability	
	RO1.4	Sleep quality		Subjective; circadian		Sleep improvement, insomnia, sleep apnea, waking rested, deep sleep, better sleep	
	RO1.5	Appetite and satiety		Subjective; metabolic		Hunger, cravings, satiety, appetite control, sugar cravings gone, no longer hungry	
	RO1.6	Pain and inflammation		Subjective; musculoskeletal		Pain, chronic pain, back pain, joint pain, headache, migraine, swelling, stiffness	
	RO1.7	Digestive health		Subjective; gastrointestinal		Bloating, IBS^b^, constipation, acid reflux, gut health, digestion improved, heartburn	
	RO1.8	Skin health		Subjective; dermatological		Acne, eczema, psoriasis, rash, skin cleared, skin tags, dermatitis, rosacea	
	RO1.9	Hormonal and menstrual health		Subjective; endocrine		Hormonal symptoms, menstrual cycle, PMS^c^, perimenopause, hot flushes, libido	
**RO2: Tool-mediated validation (8 aspects): outcomes verified through measurement tools, clinical tests, or quantification**							
	RO2.1	Anthropometric changes		Objective; measured		Weight, pounds, kg, lbs lost, waist, BMI, body fat, dress size, inches, visceral fat	
	RO2.2	Glycemic control		Objective; lab biomarker		Blood sugar, A_1C_, HbA_1c_^d^, fasting glucose, fasting insulin, CGM^e^, glucometer reading	
	RO2.3	Blood pressure		Objective; measured		Blood pressure, systolic, diastolic, BP normalized, hypertension controlled, mmHg	
	RO2.4	Lipid profile		Objective; lab biomarker		Cholesterol, triglycerides, HDL^f^, LDL^g^, lipid panel, cholesterol improved, statins off	
	RO2.5	Inflammatory markers		Objective; lab biomarker		CRP, C-reactive protein, inflammatory markers, ESR^h^, inflammation markers reduced	
	RO2.6	Liver function		Objective; lab biomarker		ALT^i^, AST^j^, liver enzymes, liver function test, fatty liver markers, liver normalized	
	RO2.7	Kidney function		Objective; lab biomarker		Creatinine, GFR^k^, eGFR^l^ improved, kidney function tests, creatinine normalized	
	RO2.8	Hormonal markers		Objective; lab biomarker		Testosterone, estrogen, thyroid (TSH/T3/T4), cortisol, hormonal lab values improved	
**RO3: Disease specificity (18 aspects): reported improvements in named medical conditions**							
	RO3.1	Type 2 diabetes		Disease; metabolic		Diabetes, diabetic, T2D, prediabetes, reversed diabetes, off metformin, off insulin	
	RO3.2	Fatty liver disease		Disease; hepatic		Fatty liver, NAFLD^m^, NASH^n^, liver disease, fatty liver reversed, hepatic steatosis	
	RO3.3	Cardiovascular disease		Disease; cardiac		Heart disease, heart failure, coronary artery disease, heart attack, cardiovascular	
	RO3.4	Hypertension		Disease; cardiovascular		Hypertension, high blood pressure, off blood pressure medication, BP controlled	
	RO3.5	PCOS		Disease; endocrine		PCOS, polycystic ovary syndrome, polycystic ovaries, PCOS symptoms improved	
	RO3.6	Neurodegenerative disease		Disease; neurological		Alzheimer, dementia, Parkinson, neurodegeneration, cognitive decline reversed	
	RO3.7	Chronic kidney disease		Disease; renal		Kidney disease, CKD, chronic kidney disease, kidney failure, renal function improved	
	RO3.8	Gout		Disease; metabolic		Gout, uric acid, gout attack gone, no more gout, uric acid normalized	
	RO3.9	Cancer		Disease; oncological		Cancer, tumor, remission, cancer markers, prostate cancer, cancer improved	
	RO3.10	Osteoporosis		Disease; musculoskeletal		Osteoporosis, bone density, bone loss, osteopenia, DEXA scan improved	
	RO3.11	Stroke		Disease; cerebrovascular		Stroke, TIA, mini stroke, stroke recovery, stroke risk reduced	
	RO3.12	ADHD		Disease; neurodevelopmental		ADHD, attention deficit, ADD, hyperactivity, ADHD symptoms improved, focus	
	RO3.13	Thyroid disease		Disease; endocrine		Thyroid, hypothyroid, hyperthyroid, Hashimoto’s, thyroid medication reduced	
	RO3.14	Inflammatory bowel disease		Disease; gastrointestinal		Crohn, ulcerative colitis, IBD, Crohn in remission, colitis improved	
	RO3.15	Autoimmune disease		Disease; immunological		Autoimmune, lupus, multiple sclerosis, rheumatoid arthritis, celiac, autoimmune improved	
	RO3.16	Fibromyalgia and neuropathy		Disease; neurological		Fibromyalgia, neuropathy, nerve pain, peripheral neuropathy, numbness, tingling gone	
	RO3.17	Arthritis		Disease; musculoskeletal		Arthritis, osteoarthritis, arthritic, rheumatoid, joint disease, arthritis improved	
	RO3.18	Gallbladder disease		Disease; biliary		Gallbladder, gallstones, cholecystectomy, gallbladder attack, gallstones resolved	

^a^Exclusion patterns redirect keyword matches to more specific aspects to prevent double-counting (eg, “arthritis” in a pain context is classified under RO3.17, not RO1.6). All keywords are matched case-insensitively with whole-word boundary constraints. Complete keyword sets and exclusion patterns are available in the supplementary materials repository.

^b^IBS: irritable bowel syndrome.

^c^PMS: premenstrual syndrome.

^d^HbA_1c_: hemoglobin A_1c_.

^e^CGM: continuous glucose monitor.

^f^HDL: high-density lipoprotein.

^g^LDL: low-density lipoprotein.

^h^ESR: erythrocyte sedimentation rate.

^i^ALT: alanine aminotransferase.

^j^AST: aspartate aminotransferase.

^k^GFR: glomerular filtration rate

^l^eGFR: estimated glomerular filtration rate.

^m^NAFLD: nonalcoholic fatty liver disease.

^n^NASH: nonalcoholic steatohepatitis.

### Ontology Validation and Refinement

The ontology underwent 2 validation rounds (coverage testing and precision refinement) before deployment. A final manual review of 20 randomly sampled matches per aspect confirmed semantic validity before the ontology was locked for Phase 3 application.

Final ontology coverage showed that 30.1% (12,976/43,111) of comments contained at least one health-relevant keyword match; of these, 4.15% (n=1790) met all criteria for a definite, first-person, positive health outcome report.

### Phase 3: Classification Framework

#### Overview

**Figure 2:**
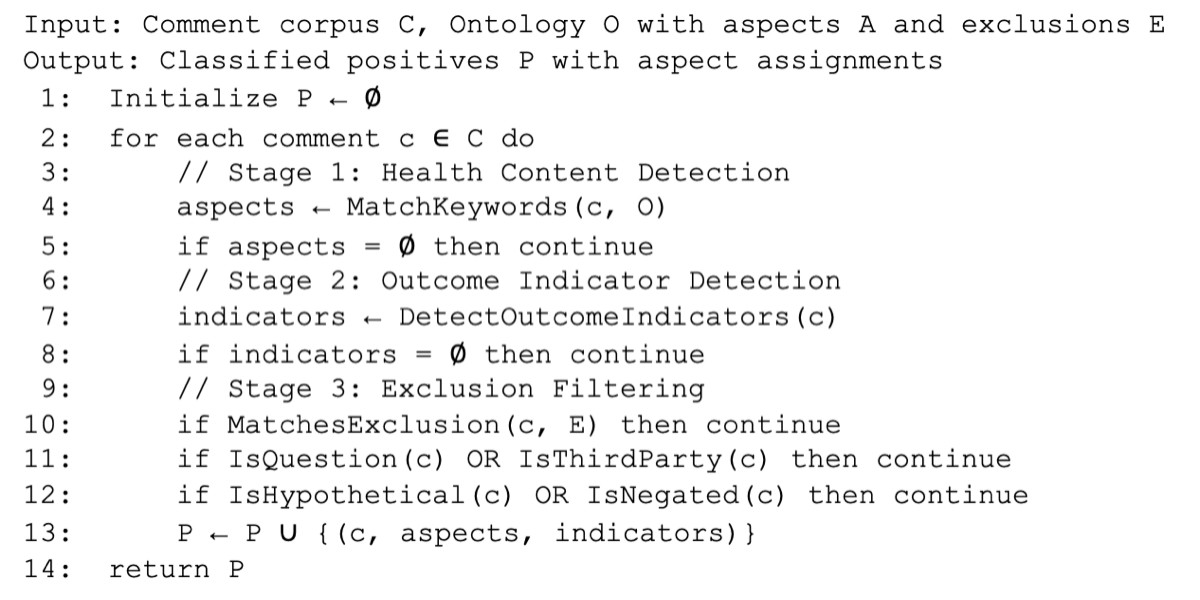
Algorithm 1: precision-optimized health outcome classification.

The classification system implements a 3-stage pipeline designed to maximize precision. Algorithm 1 in Figure 2 presents the formal classification procedure.

#### Stage 1: Health Content Detection

Comments are filtered for health-related content using keyword matching against the ontology vocabulary.

#### Stage 2: Outcome Indicator Detection

Health-related comments are analyzed for positive outcome indicators, including quantified changes (eg, “lost 30 pounds”), symptom cessation (eg, “pain gone”), explicit improvement language (eg, “reversed my diabetes”), and temporal improvement markers (eg, “no longer need medication”).

#### Stage 3: Exclusion Filtering

Candidate positives are filtered through exclusion patterns removing: (1) questions rather than statements, (2) third-party reports, (3) hypothetical or aspirational language, (4) negated outcomes, and (5) general health advice. This conservative approach implements the precision-optimized design philosophy.

Handling of ambiguous statements: Sarcasm is not specifically detected but is unlikely to pass all 3 stages due to the requirement for co-occurring health vocabulary, outcome indicators, and absence of exclusion patterns. Implied improvements without explicit outcome language are intentionally excluded. Narrative sequences mixing positive and negative outcomes may partially pass but are addressed by negation filters. The validation results confirm that such cases account for a minority of false positives (complete classification rule patterns are provided in Multimedia Appendix 4).

### Validation Studies

#### Overview

The classification framework was stress-tested through 5 complementary validation studies, each probing a different dimension of performance: internal precision on the development corpus, recall and coverage through weighted negative sampling, out-of-sample generalizability on held-out channels, rater agreement via LLM-assisted interrater reliability assessment, and head-to-head comparison against fine-tuned transformer baselines. Each study is described in turn below; full protocols and results for the external validation and transformer baseline are reported in Multimedia Appendices 5 and 6, respectively.

#### Precision Validation

Classification precision was validated through manual coding of a stratified random sample. Sample size was calculated for 95% confidence level with 4% margin of error, yielding n=500 samples stratified by RO. Each sample was coded on five dimensions: (1) is_positive_outcome (yes, no, or unclear), (2) is_personal (yes or no), (3) is_definite (yes or no), (4) aspect_correct (yes, partial, or no), and (5) free-text notes. Wilson score [44] confidence intervals were computed for all proportions.

#### Recall Estimation

Recall was estimated through stratified negative sampling. A stratified random sample of n=510 comments from the nonclassified pool (41,321 comments not identified as positive outcomes) was drawn using disproportionate allocation across 3 comment-length strata (short: fewer than 50 words, n=300; medium: 50 to 150 words, n=150; long: more than 150 words, n=60) with proportional allocation across the 11 channels within each stratum. Each sampled comment was manually reviewed by the first author to identify false negatives: true positive outcomes missed by the classification system. Sampling weights reflecting the population proportion in each length stratum were applied to compute unbiased weighted false-negative rates, which were then extrapolated to estimate total missed positives and to calculate recall. Wilson score CIs were used throughout.

#### External Validation on Held-Out Channels

To address the concern that the 35-aspect ontology was iteratively refined on the same corpus used for classification, and thereby to test generalizability beyond the development channels, external validation was conducted on 12,653 comments collected from 5 independent YouTube channels with zero overlap with the development corpus. The 5 held-out channels (Georgia Ede MD, Robert Kiltz MD, Sten Ekberg DC, Chris Palmer MD, and Ted Naiman MD) were selected by an independent co-author based on topical relevance to ketogenic, carnivore, and broader metabolic health dietary content. For each channel, comments were collected from the 10 most-commented videos using the YouTube data API, following the same protocol as the development corpus. The classifier was applied without modification. All comments classified as positive health outcomes were exhaustively verified through manual coding to determine precision (census approach). For recall estimation, a stratified random sample of nonpositive comments (up to 100 per channel; random seed 42) was manually coded to identify false negatives, with channel-level false-negative rates extrapolated to the full negative population, and 95% Wilson score CIs reported. The full external validation protocol and results are presented in Multimedia Appendix 5.

#### Interrater Reliability Assessment

To address the limitation of single-coder validation, we used an LLM-assisted annotation validation protocol using 2 independent LLM coders (GPT-4o and GPT-4.1) (OpenAI) as second annotators [45,46]. This approach, increasingly adopted in computational linguistics and health informatics, provides a systematic assessment of interrater reliability while maintaining full reproducibility.

From the 500-sample precision validation set, 28 exemplars were selected through purposive stratified sampling to serve as few-shot coding examples. The stratification covered 6 coding outcome categories: clear true positives (n=10), clear negatives or false positives (n=5), unclear or ambiguous cases (n=5), positive but not personal outcomes (n=3), positive but not definite outcomes (n=2), and aspect assignment issues (n=3). This selection ensured representation of all 3 ROs, 10 of 11 channels, all 6 outcome categories, and deliberate overrepresentation of minority classes and boundary cases, which is standard practice in few-shot prompt design.

Both models received identical structured prompts containing: (1) the coding task definition with detailed guidelines for all four coding dimensions, (2) the complete 35-aspect ontology reference, (3) all 28 few-shot exemplars with the researcher’s ground-truth codings, and (4) the comment text with automated classification details but without the researcher’s manual codings (full prompt provided in Multimedia Appendix 6). Processing used temperature 0.0 (deterministic output) in batches of 10 comments. Agreement was computed on the remaining 472 test samples (exemplars excluded to prevent circular validation).

To mitigate anchoring effects, exemplars spanned 6 coding outcome categories (including clear negatives and errors), and the prompt instructed models to code independently based on the comment text, explicitly stating that the automated classification may be incorrect.

Cohen κ [47] was computed for each coding dimension across 3 comparison pairs (human vs GPT-4o, human vs GPT-4.1, and GPT-4o vs GPT-4.1). Because the validation set’s 90.4% positive class prevalence creates a κ paradox [48,49], we report the prevalence index and bias index alongside each κ value to decompose the paradox. Raw percent agreement and Cohen κ are the primary reliability metrics, interpreted using the Landis and Koch [47] framework.

#### Transformer Baseline Comparison

To test whether the precision advantage of the rule-based framework comes at an unnecessary cost to recall compared with learned representations trained on the same data, a head-to-head baseline comparison was conducted against two pretrained transformer models: Bidirectional Encoder Representations from Transformers (BERT)-base-uncased and Robustly Optimized BERT Pretraining Approach (RoBERTa)-base (Hugging Face). Both models were fine-tuned on the combined precision-validation and recall-expansion datasets (n=836 unique manually-coded comments; 347 positive and 489 negative) using stratified 5-fold cross-validation. Standard pretrained weights and default fine-tuning hyperparameters were used; no hyperparameter search was performed, because the purpose of this analysis was a fair comparison against a reasonable learned baseline rather than an optimized benchmark. Performance was evaluated on 4 metrics (precision, recall, *F*_1_-score, and receiver operating characteristic-area under the curve) averaged across folds, and, separately, on the 326-comment precision-validation subset and on the 27 false negatives identified through the recall-expansion sample. The rule-based framework was compared against both transformer baselines on each metric. The full experimental protocol, hyperparameters, and per-fold results are presented in Multimedia Appendix 7.

### Supplementary Analysis: Aspect-Based Sentiment Contextualization

This supplementary analysis is reported here, rather than as a coequal phase of the primary methodology, for 2 reasons. First, it uses a methodologically distinct procedure (LLM consensus coding) that cannot be directly compared to the validation regime applied to the rule-based framework. Second, its purpose is to explore and contextualize the positive-only design of the primary framework, not to estimate the prevalence of negative outcomes in the underlying population. Claims derived from this analysis are therefore treated as indicative rather than confirmatory throughout the Discussion section.

Because the classification framework extracts only positive health outcomes (as outlined in the Research Design Overview section), a supplementary analysis was conducted to contextualize this scope decision by characterizing the broader sentiment landscape of the corpus. ABSA was used to quantify the distribution of positive, negative, neutral, and mixed health sentiment across comments, providing an empirical basis for evaluating whether the positive-only focus omits a substantial volume of negative health experiences.

ABSA extends document-level sentiment analysis by identifying specific aspects (topics or entities) within a text and assigning sentiment to each aspect independently [50,51]. This granularity is essential for health-related comments, which frequently contain mixed sentiment. For example, a single comment reporting weight loss improvement alongside gastrointestinal discomfort.

A stratified random sample of 1000 comments was drawn from the full corpus, proportional to channel contribution. Two independent LLMs (GPT-4o and GPT-4.1) were prompted to perform ABSA on each comment, classifying it as health-related or nonhealth-related and, for health-related comments, identifying health aspects and assigning aspect-level sentiment (positive, negative, neutral, or mixed). The dual-model design serves as a form of interrater reliability assessment: only comments in which both models agree on health-relatedness and sentiment classification are included in the consensus analysis, yielding conservative yet high-confidence sentiment estimates. The complete ABSA prompt, including the task definition, coding guidelines, and a few-shot exemplar, is provided in Multimedia Appendix 8.

## Results

### Classification Performance

The framework classified 1790 comments (1790/43,111, 4.15% of the corpus) as containing definite positive health outcomes. Table 3 presents the complete validation metrics.

**Table 3:** Classification validation results for a rule-based natural language processing framework applied to English-language YouTube comments on 11 metabolic health channels (N=43,111 unique comments, November 2013 to January 2026). Precision validated on n=500 stratified random samples; recall estimated from n=510 stratified negative samples. Wilson score 95% CIs.

Metric	Value	95% CI (%)
Precision, % (n=500)	97.6	95.7-98.6
Recall, % (n=510)	56.2	43.4-67.9
*F*_1_-score, %	28.3	—^a^
True positives (validated)	488/500	—
False positives	11/500	—
False negatives (in sample)	27/510	—
First-person testimony rate, %	97	95.2-98.1
Definite outcome rate, %	88.4	85.3-90.9
Aspect assignment accuracy (strict), %	90.8	88-93
Aspect assignment accuracy (lenient), %	97	95.2-98.1

^a^Not applicable.

The expanded recall estimation (n=510 stratified sample) identified 27 false negatives (5.3% raw rate; 3.29% weighted rate), yielding an estimated recall of 56.2% (95% CI 43.4%-67.9%) when extrapolated to the full nonpositive pool. False negatives varied across channels (χ²_10_=28.8; *P*=.001) and comment length strata (χ²_2_=19.4; *P*<.001), with KenDBerryMD (16.7%) and Eric Berg DC (11.4%) showing the highest channel rates and medium-length (10%) and long (11.7%) comments generating more false negatives than short comments (1.7%). The dominant miss reason was structural pattern mismatch (23 of 27, 85%), indicating that the classifier keyword dictionary is adequate, but its syntactic pattern set does not capture all expression forms. Applying precision estimates to the classified corpus yields 1747 estimated true-positive health outcome reports (95% CI 1713-1764).

### Error Analysis

To characterize the framework’s failure modes, we examined all false positives from the precision validation (n=11 across 9 unique comments) and all false negatives from the recall estimation (n=27). Three systematic categories of false positive errors were identified: third-party outcome references (4 of 11 cases, 36%), where comments described health improvements experienced by family members or acquaintances rather than the commenter; negative overall trajectory contexts (4 of 11, 36%); and nonspecific or advice-based language (3 of 11, 27%).

Among false positives, the dominant pattern was positive signals embedded in negative overall trajectories (4/11, 36%), followed by nonspecific or advice-based language (3/11, 27%) and third-party reports (2/11, 18%). These cases require discourse-level sentiment analysis beyond the current sentence-level pattern matching.

False negative analysis revealed structural pattern mismatch as the dominant miss mechanism (23/27, 85%), indicating that the keyword dictionary is adequate, but syntactic patterns do not capture all expression forms. The most frequently missed aspects were RO2.1 (general well-being, 17/27), RO1.8 (energy, 6/27), and RO1.2 (body composition, 5/27). Targeted syntactic rule expansion, rather than vocabulary expansion, represents the primary pathway to improved recall (Table 4).

**Table 4:** Error analysis for a rule-based natural language processing framework classifying self-reported health outcomes in English-language YouTube comments on metabolic health channels (N=43,111 unique comments, November 2013 to January 2026). False positives (n=11) came from precision validation of 500 samples; false negatives (n=27) came from recall estimation of 510 samples.

Type	Category	Errors, n (%)	Suggested remedy
FP^a^	Third-party references	4 (36)	Expand person-reference filters
FP	Negative trajectory context	4 (36)	Discourse-level sentiment analysis
FP	Nonspecific or advice language	3 (27)	Tighter personal experience requirements
FN^b^	Borderline or debatable positives	7 (26)	Broader outcome definitions (recall trade-off)
FN	Colloquial symptom language	10 (37)	Vocabulary expansion
FN	Implicit emotional language	8 (30)	ML^c^-based semantic classification
FN	Missed pattern coverage	2 (7)	Rule refinement

^a^FP: false positive; n=11.

^b^FN: false negative; n=27.

^c^ML: machine learning.

External validation was conducted on 12,653 comments from 5 YouTube channels not included in the development corpus, selected by an independent co-author. The classifier achieved 93.4% precision (227/243; 95% CI 89.6%-95.9%) on the external corpus, with CIs overlapping those from the development corpus (97.6%), confirming generalizability within the metabolic health domain. Recall was estimated at 50.1% (95% CI 31.4%-59.1%), consistent with the development corpus. Full external validation protocol and results are presented in Multimedia Appendix 5.

### Interrater Reliability

Table 5 presents the interrater reliability results across 4 coding dimensions and 3 comparison pairs, with raw Cohen κ and percentage agreement as primary metrics.

**Table 5:** Interrater reliability for validation of a rule-based natural language processing framework classifying self-reported health outcomes in English-language YouTube comments on 11 metabolic health channels (N=43,111 unique comments, November 2013 to January 2026). Reliability was assessed across four coding dimensions and three comparison pairs. Cohen κ and percent agreement are reported as primary metrics. Prevalence index (PI) [49] and bias index (BI) [52] quantify the κ paradox components; Κ interpretation follows Landis and Koch [47]. The validation set was drawn from classifier-positive comments, producing approximately 90% positive prevalence that depresses κ values via the κ paradox [48,49].

Comparison	Dimension	Agree, n (%)	Cohen κ	Interpretation	PI^a^	BI^b^
Human vs GPT-4o	Positive health outcome	394/471 (83.7)	0.297	Fair	0.892	0.076
Human vs GPT-4o	First-person testimony	463/472 (98.1)	0.658	Substantial	0.962	0.019
Human vs GPT-4o	Definite outcome	316/472 (66.9)	0.130	Slight	0.788	0.242
Human vs GPT-4o	Aspect assignment	289/472 (61.2)	0.106	Slight	0.896	0.326
Human vs GPT-4.1	Positive health outcome	410/471 (87)	0.332	Fair	0.892	0.045
Human vs GPT-4.1	First-person testimony	468/471 (99.4)	0.839	Almost perfect	0.966	0.006
Human vs GPT-4.1	Definite outcome	389/472 (82.4)	0.205	Slight	0.788	0.040
Human vs GPT-4.1	Aspect assignment	267/472 (56.6)	0.082	Slight	0.896	0.373
GPT-4o vs GPT-4.1	Positive health outcome	445/472 (94.3)	0.771	Substantial	0.767	0.044
GPT-4o vs GPT-4.1	First-person testimony	463/472 (98.1)	0.706	Substantial	0.928	0.013
GPT-4o vs GPT-4.1	Definite outcome	375/472 (79.4)	0.476	Moderate	0.305	0.201
GPT-4o vs GPT-4.1	Aspect assignment	385/472 (81.6)	0.655	Substantial	0.523	0.072

^a^PI: prevalence index.

^b^BI: bias index.

On the primary coding dimension (positive health outcome identification), raw percent agreement was high (83.7%-94.3%), but Cohen κ values were lower (0.297-0.771), reflecting the well-documented κ paradox [48,49]: the validation set’s 90.4% positive prevalence leaves limited headroom for κ above chance. Bias Index values (0.044-0.076) confirm that low κ is driven by prevalence rather than systematic rater bias.

For personal testimony identification, agreement was near-ceiling across all pairs. GPT-4.1 achieved the highest κ observed in this study (κ=0.839, 99.4% agreement), while GPT-4o achieved substantial agreement (κ=0.658, 98.1%). The lower κ for GPT-4o, despite 98.1% agreement, again reflects the prevalence paradox: with 98% of comments coded as first-person testimony, κ is constrained even at very high observed agreement.

On secondary dimensions (definiteness and aspect correctness), agreement was lower, with systematic directional bias: both LLMs applied stricter evidentiary standards than the human coder (McNemar *P*<.001 and *P*=.048), consistent with measurement bias characterized in the LLM Bias Audit (Multimedia Appendix 9).

The cross-model comparison provides the strongest reliability evidence: two independent architectures achieved substantial agreement on positive outcome identification (κ=0.771, 94.3%), the highest human-level or cross-model κ for this dimension. This cross-architecture convergence suggests that the coding task is well-specified: two independent systems, given the same instructions, reach similar conclusions. The cross-model κ is less affected by the prevalence paradox because both models have more balanced marginal distributions than the human-vs-LLM comparisons (PI=0.767 vs 0.892).

### Prevalence and Distribution by Research Objective

The raw prevalence of classified positive outcomes was 4.15% (1790/43,111). Adjusted for precision, the estimated true positive prevalence is 4.05%. Outcomes were distributed across ROs as shown in Table 6. Of these, 50.3% of positive outcome comments (n=3355) spanned multiple ROs, indicating users frequently report improvements across multiple health dimensions simultaneously.

**Table 6:** Distribution of positive health outcomes by research objective, extracted from English-language YouTube comments on 11 metabolic health channels using a rule-based natural language processing framework (n=6674 positive outcomes in n=1790 positive reports among N=43,111 unique comments, November 2013 to January 2026). Percentages sum to more than 100% because 50.3% of outcomes span multiple research objectives. Wilson score 95% CIs.

RO^a^	Description	Positive outcomes (n=6674), n (%; 95% CI)
RO1	Subjective well-being	3456 (51.8; 50.6-53)
RO2	Tool-mediated validation	5350 (80.2; 79.2-81.1)
RO3	Disease specificity	2032 (30.5; 29.4-31.6)

^a^RO: research objective.

### Health Aspect Analysis

Table 7 presents the top 10 health aspects by frequency. Anthropometric changes (primarily weight loss) dominated at 73% (4870/6674) of positive outcomes, consistent with the metabolic health focus of the source content. Pain and inflammation reduction (1137/6674, 17%) and type 2 diabetes improvement (977/6674, 14.6%) were the second and third most reported outcomes, suggesting clinically significant health impacts beyond aesthetic weight changes.

**Table 7:** Top 10 most frequently reported health aspects among self-reported positive outcomes extracted from English-language YouTube comments on 11 metabolic health channels (n=1790 reports of 6674 positive outcomes among N=43,111 unique comments, November 2013 to January 2026). Percentages computed relative to total positive outcomes. Wilson score 95% CIs.

#	ID	Aspect	Positive outcomes, n (%; 95% CI)
1	RO^a^ 2.1	Anthropometric changes	4870 (73; 71.9-74.1)
2	RO1.6	Pain and inflammation	1137 (17; 16.2-18)
3	RO3.1	Type 2 diabetes	977 (14.6; 13.8-15.5)
4	RO1.8	Skin health	784 (11.8; 11-12.5)
5	RO1.3	Psychological well-being	731 (11; 10.2-11.7)
6	RO1.5	Appetite and satiety	677 (10.1; 9.4-10.9)
7	RO1.7	Digestive health	664 (10; 9.3-10.7)
8	RO1.2	Energy and vitality	651 (9.8; 9.1-10.5)
9	RO2.2	Glycemic control	564 (8.5; 7.8-9.1)
10	RO2.3	Blood pressure	548 (8.2; 7.6-8.9)

^a^RO: research objective.

### Channel-Level Variation

Significant variation in positive outcome rates was observed across channels (χ²₁₀=927.5; *P*<.001), as shown in Table 8. Rates ranged from 1.32% (Shawn Baker, MD) to 10.40% (KenDBerryMD), yielding an odds ratio of 8.68 between the highest and lowest channels. Cramér *V*=0.147 indicates a small but statistically significant effect size, suggesting that while channel-level differences exist, they explain a modest proportion of total variance in outcome reporting.

**Table 8:** Channel-level variation in positive health outcome rates in a cross-sectional computational analysis of self-reported outcomes from English-language YouTube comments on 11 metabolic health channels (N=43,111 unique comments, November 2013 to January 2026). Channels ordered by descending positive outcome rate. Wilson score 95% CIs.

Channels	Positive outcomes, n/N (%; 95% CI)
KenDBerryMD	413/3970 (10.40; 9.49-11.39)
Jason Fung	286/3963 (7.22; 6.45-8.06)
Eric Berg DC	282/3993 (7.06; 6.31-7.90)
Eric Westman	250/3978 (6.28; 5.57-7.08)
Anthony Chaffee MD	104/3972 (2.62; 2.17-3.16)
Dr. Robert Cywes MD	102/3932 (2.59; 2.14-3.14)
Dr. Boz	86/3959 (2.17; 1.76-2.67)
Nick Norwitz	82/3954 (2.07; 1.67-2.57)
Ben Bikman	65/3477 (1.87; 1.47-2.38)
Mark Hyman	68/3980 (1.71; 1.35-2.16)
Shawn Baker MD	52/3933 (1.32; 1.01-1.73)
Overall	1790/43,111 (4.15; 3.96-4.35)

### Outcome Category Distribution

Analysis of outcome indicator types (Table 9) revealed that quantified changes (eg, “lost 30 pounds,” “A1C dropped to 5.4”) comprised 74.4% (1331/1790) of positive outcomes. Symptom cessation reports (eg, “joint pain gone”) accounted for 14.5% (259/1790), explicit improvement language (eg, “feel so much better”) for 11.8% (212/1790), and disease reversal or remission claims (eg, “reversed my type 2 diabetes”) for 6.4% (114/1790). Medication discontinuation (eg, “off all medications”) represented 3.9% (69/1790), and temporal improvements (eg, “since starting keto…lost 20 pounds”) represented 2.3% (42/1790) of outcomes reported.

**Table 9:** Distribution of outcome indicator categories in a cross-sectional computational analysis of self-reported health outcomes from English-language YouTube comments on 11 metabolic health channels (n=1790 positive outcomes from N=43,111 unique comments, November 2013 to January 2026). Categories are not mutually exclusive; a single comment may contain multiple indicator types.

Outcome category	Positive outcomes, n (%)
Quantified change	1331 (74.4)
Symptom cessation	259 (14.5)
Explicit improvement	212 (11.8)
Reversal or remission	114 (6.4)
Medication discontinuation	69 (3.9)
Temporal improvement	42 (2.3)

### Sentiment Contextualization: ABSA

A supplementary ABSA was conducted to contextualize the positive-outcome findings within the broader health discourse of the corpus [50,51].

Intermodel agreement on health-related classification was 93.1% (915/983), with sentiment agreement of 87.6% (495/565), indicating acceptable coding consistency for an exploratory contextualization analysis.

Table 10 presents the consensus sentiment distribution, the subset of health-related comments where both models agreed on sentiment classification. Among 495 consensus-coded health-related comments, positive sentiment accounted for 54.7% (271/495), negative for 11.9% (59/495), neutral for 15.6% (77/495), and mixed for 17.8% (88/495), yielding a positive-to-negative ratio of 4.6:1.

**Table 10:** Aspect-based sentiment analysis (ABSA) consensus sentiment distribution among health-related English-language YouTube comments on 11 metabolic health channels (n=495 consensus-coded comments from a stratified sample of 1003 drawn from N=43,111 unique comments, November 2013 to January 2026). Dual-model classification (GPT-4o and GPT-4.1) with consensus defined as agreement on both health-relatedness and sentiment polarity.

Sentiment^a^	Comments, n (%)	Estimated full corpus
Positive	271 (54.7)	~63,800
Negative	59 (11.9)	~13,900
Neutral	77 (15.6)	~18,200
Mixed	88 (17.8)	~20,800

^a^Consensus: both GPT-4o and GPT-4.1 agreed on sentiment classification. Corpus estimates extrapolated from 64.4% health-related rate in the stratified sample (n=983). Positive-to-negative ratio=4.6:1.

Table 11 presents the breakdown of the consensus-negative aspect. Gastrointestinal issues (n=36) and cardiovascular concerns (n=22, primarily LDL cholesterol elevations) were the most frequent negative aspects, followed by pain and inflammation (n=14) and energy and mood disturbances (n=12), aligning with documented adaptation effects during carbohydrate restriction transitions.

**Table 11:** Consensus-negative health aspects identified by dual-model aspect-based sentiment analysis (ABSA) of English-language YouTube comments on 11 metabolic health channels (N=43,111 unique comments, November 2013 to January 2026). Both GPT-4o and GPT-4.1 agreed on negative sentiment classification for each aspect listed.

Health aspect	Comments reaching consensus^a^, n	Clinical context
Digestive	36	GI^b^ adaptation
Cardiovascular	22	LDL^c^ concerns
Pain and inflammation	14	Adaptation effects
Energy and mood	12	Transition fatigue
Neurological	12	Keto adaptation
Blood sugar	11	Glycemic worsening
Weight change	11	Weight stall or gain
Other (sleep, diet adherence, mental health, medication, general well-being, cancer, skin, autoimmune, and hormonal)	63	Various

^a^Consensus-negative: both GPT-4o and GPT-4.1 independently classified the aspect sentiment as negative. Total exceeds 59 comments because some comments contain multiple negative aspects.

^b^GI: gastrointestinal.

^c^LDL: low-density lipoprotein.

The 4.6:1 positive-to-negative ratio in this sample is consistent with the expected self-selection dynamics of the channels studied, while the presence of negative experiences at a meaningful rate suggests that the positive predominance of the primary framework is not solely an artefact of its positive-only scope. This finding is exploratory: generalization to the full corpus would require applying the same rigor used for the primary framework.

Table 12 presents aspect-level ratios, revealing that the positive predominance is not uniformly distributed. Weight change (8.9:1) and general well-being (7.1:1) exhibit the strongest positive skew, while digestive health (0.8:1), neurological symptoms (0.5:1), and hormonal concerns (0.3:1) are negative-dominant, reflecting known adaptation effects. The cardiovascular domain shows near-parity (1.0:1), consistent with the contested nature of LDL cholesterol responses to high-fat diets.

**Table 12:** Aspect-level sentiment distribution by health domain in English-language YouTube comments on 11 metabolic health channels (GPT-4.1 classification, n=627 health-related comments from a stratified sample of 1003 drawn from N=43,111 unique comments, November 2013 to January 2026). Positive-to-negative ratio computed for aspects with at least 5 negative mentions.

Health aspect^a^	Positive, n	Negative, n	Neutral, n	Mixed, n	Positive to negative ratio	Negative, n/N (%)
Weight change	241	27	27	15	8.9:1	27/310 (8.7)
General well-being	213	30	13	2	7.1:1	30/258 (11.6)
Skin	21	5	1	0	4.2:1	5/27 (18.5)
Medication	30	10	34	2	3.0:1	10/76 (13.2)
Energy and mood	52	18	1	3	2.9:1	18/74 (24.3)
Blood sugar	51	19	45	1	2.7:1	19/116 (16.4)
Diet adherence	96	38	60	8	2.5:1	38/202 (18.8)
Sleep	22	9	1	0	2.4:1	9/32 (28.1)
Pain and inflammation	54	26	5	0	2.1:1	26/85 (30.6)
Cardiovascular	33	32	41	6	1.0:1	32/112 (28.6)
*Digestive*	46	56	27	2	0.8:1	56/131 (42.7)
*Mental health*	7	11	1	0	0.6:1	11/19 (57.9)
*Neurological*	7	15	3	0	0.5:1	15/25 (60.0)
*Hormonal*	2	6	8	1	0.3:1	6/17 (35.3)

^a^Italicized aspects are negative-dominant (ratio <1.0), that is, the percentage of all mentions within that aspect classified as negative. GPT-4.1 single-model classification (n=627 health-related comments, n=1602 total aspect mentions).

To contextualize the rule-based framework’s performance, BERT-base-uncased and RoBERTa-base classifiers were trained on the combined validation datasets (n=836; using a five-fold stratified cross-validation). Both transformer models achieved substantially higher recall (93.4% and 95.7%) but lower precision (87% and 88.2%) than the rule-based framework (97.6%), confirming the design advantage of precision optimization for high-confidence corpus generation. Full transformer baseline comparison results are presented in Multimedia Appendix 7.

Table 13 positions this work relative to prior approaches, with the key differentiation being explicit precision optimization for high-confidence corpus generation.

**Table 13:** Comparison of the proposed rule-based natural language processing framework with prior social media health text classification approaches. The proposed framework was applied to English-language YouTube comments on 11 metabolic health channels (N=43,111 unique comments, November 2013 to January 2026). Precision and recall are reported for the positive class (health event).

Study	Platform	Approach	Precision	Recall	Ontology
Sarker and Gonzalez [53]	Twitter	ML^a^ (SVM)^b^	85%	82%	ADR-based
Nikfarjam et al [30]	Twitter	CRF^c^	87%	71%	UMLS
Golder et al [43]	Multi	ML (Review)	80-90%	Varies	Varies
Magge et al [54]	Twitter	DL^d^ (RoBERTa)	63%^e^	63%^e^	MedDRA
This study	YouTube	Rule-based	97.6%	56.2%	Custom

^a^ML: machine learning.

^b^SVM: support vector machine.

^c^CRF: conditional random field.

^d^DL: deep learning.

^e^Evaluated at a realistic 7% positive rate. At 2% positive rate (comparable to this study’s 4.15%), precision dropped to 21% and recall to 25%.

## Discussion

### Principal Findings

This study set out to determine whether self-reported positive health outcomes can be systematically extracted from YouTube comments on metabolic health healthcasting channels, and, if so, to characterize their prevalence, distribution across health aspects, variation across content creators, and the classification accuracy required to generate a validated corpus.

#### RQ1: What Is the Prevalence of Self-Reported Positive Health Outcomes in YouTube Comments on Metabolic Health Content?

The classification framework identified 1790 definite positive self-reported health outcome comments from a corpus of 43,111 unique comments across eleven metabolic health healthcasting channels, corresponding to a raw prevalence of 4.15%. These reports are unsolicited first-person accounts of health improvement, posted spontaneously under creator videos rather than in response to surveys or prompts, making them a distinctive source of real-world health data. The observed prevalence remained stable during external validation on an independent set of 5 held-out channels, suggesting that positive outcome reporting occurs at a consistent, detectable frequency across healthcasting communities in this domain. A supplementary sentiment analysis of the broader health discourse confirmed that positive outcome reporting constitutes a substantial but not overwhelming share of health-related conversation, with a positive-to-negative ratio of approximately 4.6:1.

#### RQ2: What Types of Health Outcomes Are Most Frequently Reported, and How Are They Distributed Across Subjective, Objective, and Disease-Specific Categories?

The outcome landscape that emerged from the corpus was considerably broader than the weight-loss framing commonly associated with carbohydrate restriction [3,5]. Positive outcomes were systematically organized using a newly developed 35-aspect hierarchical ontology aligned with 3 complementary ROs: subjective well-being (how people feel day to day), objectively measurable changes (such as blood glucose and body composition), and named disease-specific improvements. Over half of positive outcome comments described improvements across multiple health dimensions, suggesting that commenters experience and report broad, interconnected health changes rather than isolated improvements. While changes in weight and body composition dominated, the most frequently reported outcomes also included reductions in pain and inflammation, improvements in glycemic control, better skin health, enhanced psychological well-being, and improved regulation of appetite, digestive health, and energy levels. Reports also identified improvements or remission across eighteen medical conditions, including type 2 diabetes (often described as reversed or resolved), fatty liver disease, hypertension, and polycystic ovary syndrome. Most reports described quantified changes (such as specific weight loss or improved blood markers), though symptom cessation, explicit improvement language, and disease reversal or remission claims were also common. Notably, medication discontinuation reports were present in the corpus, indicating that some commenters stopped prescription medication in connection with dietary changes described in creator content. The supplementary sentiment analysis added nuance to these findings: while weight change and general well-being showed strong positive skew, the digestive, neurological, and hormonal domains showed more negative than positive reports, consistent with known adaptation effects during transitions to carbohydrate restriction [17]. This finding confirms that negative health experiences are present in these communities at a meaningful rate, providing important context for interpreting the positive outcomes identified by the primary framework.

#### RQ3: Does Positive Outcome Reporting Vary Significantly Across Content Creators, and What Factors May Explain This Variation?

Positive outcome reporting was not uniformly distributed across the eleven channels, with rates ranging from 1.32% to 10.40%, a nearly ninefold difference that suggests a structural rather than a random pattern. Four channels clustered at substantially higher rates (KenDBerryMD, Jason Fung, Eric Berg DC, and Eric Westman), while the remaining 7 fell below 3%. Because comment volume was balanced across channels, this heterogeneity cannot be attributed to differences in sample size, and the pattern persisted in the external validation. The data suggest that this variation aligns with differences in creator discourse style: channels whose creators adopted accessible, user-facing communication tended to elicit longer average comments and higher testimonial rates, with outcome reports spanning a broad range of health aspects and frequently including narratives of symptom cessation and disease reversal. In contrast, channels with more scientifically oriented discourse produced fewer testimonials overall, but those that did appear were notably longer and more detailed, with higher rates of reports of medication discontinuation and outcomes spanning multiple health dimensions simultaneously. This influence extended to comment depth itself, as false-negative rates were significantly higher among medium-length and long comments than among short ones, suggesting that the more elaborate forms of self-disclosure encouraged by certain creator styles also produce more complex expressions of health outcomes that are harder for rule-based systems to capture. Together, these findings indicate that channel-level characteristics, including content style, creator approach, and community norms, meaningfully shape not only the volume but also the depth and nature of testimonial discourse within healthcasting environments.

#### RQ4: Can a Precision-Optimized Rule-Based Framework Achieve Sufficient Classification Accuracy for Generating Validated Health Outcome Corpora From User-Generated Content?

The 3-stage rule-based classifier achieved 97.6% precision in the development corpus, and this level held up under external validation across 5 held-out channels, with overlapping confidence intervals, confirming that the framework generalizes beyond the data on which it was built. Estimated recall was 56.2%, reflecting the deliberate design choice to prioritize the validity of every included case over exhaustive detection: the system captures roughly half of all positive outcome reports, but nearly everyone it identifies is correct. This trade-off yielded a validated corpus of health outcome reports at scale without manual review, demonstrating that the framework can generate research-ready datasets from large comment corpora. To assess whether the classification task itself is well defined rather than dependent on a single coder’s judgment, 2 independent AI systems (GPT-4o and GPT-4.1) were given the same coding instructions and reached substantial agreement on core outcome identification, with the strongest reliability emerging when the 2 models were compared directly to each other rather than to the human coder. A head-to-head comparison with fine-tuned deep learning models (BERT and RoBERTa) confirmed that the precision advantage is built into the rule-based design: both models achieved higher recall but lower precision, meaning that for every gain in detection coverage, a substantial number of incorrect classifications would be introduced into the corpus. Error analysis revealed that most missed cases occurred not because the framework lacked relevant health keywords, but because commenters expressed their outcomes in sentence structures the rules did not anticipate. This suggests that expanding the range of recognized expression patterns, rather than adding new terminology, is the most direct route to improved recall. Together, these results establish the framework as a reliable, fully transparent, and reproducible methodology for extracting self-reported health outcomes from unstructured user-generated content at scale, with every classification decision traceable to specific rules.

### Implications for Research and Practice

Computational infodemiology has increasingly established social media as a valuable source of real-world health data, yet systematic extraction methodologies have focused almost exclusively on pharmacovigilance and adverse-event detection from microblogging platforms [40,55,56], whereas qualitative research on online health communities has examined forum-based and support-group settings [57]. A substantial and growing body of health-related discourse exists in a setting that has received comparatively little methodological attention: the comment sections of expert-led YouTube health channels, where tens of thousands of individuals respond to long-form, creator-led content with first-person accounts of health changes [58,59]. Our results demonstrate that this content layer carries health signal at a density sufficient for systematic computational extraction, establishing healthcasting as a distinct empirical setting for health informatics research. For infodemiology and digital health researchers, this offers a complementary observational channel that captures a population segment (individuals who self-direct dietary interventions informed by credentialed online content) largely invisible to clinical registries and pharmacovigilance systems [29]. The replicable 3-phase construction workflow, hierarchical ontology, and multistudy validation flow presented in this study provide a methodological template that research groups can adapt to adjacent domains, including cardiometabolic disease, chronic pain, and mental health [60,61].

For clinical researchers and public health practitioners, understanding the health changes patients experience and report outside clinical settings is critical for designing patient-reported outcome measures, identifying underrecognized treatment effects, and monitoring population-level engagement with dietary interventions [62,63]. Our analysis reveals a self-reported outcome landscape that extends beyond the weight-loss framing commonly associated with carbohydrate restriction [3,5]. Reductions in pain and inflammation, improvements in type 2 diabetes, skin health, and psychological well-being were prominently reported, consistent with the clinical trial literature documenting multi-system effects of metabolic interventions [1,64,65]. Reports covered eighteen named disease conditions, and over half addressed multiple ROs simultaneously, suggesting that commenters experience and report systemic rather than isolated health changes. These convergences between self-reported online data and published clinical evidence identify patient-reported outcome dimensions that merit prospective investigation using designs appropriate to each dimension [62]. Perhaps most important for clinical practice, medication-discontinuation reports were present in this corpus, raising questions about patients stopping prescription therapy influenced by creator-led online content without direct medical oversight. This finding underscores the need for structured dialogue between healthcasting communities and the clinical care system to ensure that patient-initiated medication changes occur safely [63,66,67].

As digital platforms become the primary channels through which patients encounter health information and make health decisions, understanding how platform features and community dynamics shape health discourse is an increasingly important priority for health communication researchers and platform designers [20,40]. Our analysis reveals that positive outcome reporting rates vary substantially across channels operating within the same broad dietary domain, and that this variation is structurally predictable from content style and community culture rather than random. We offer interpretive hypotheses, framed as requiring systematic testing rather than as validated findings [57]: channels whose creators explicitly invite health testimonials and attract audiences with active metabolic conditions foster communities where outcome-sharing functions as a social norm, whereas science-focused channels attract audiences that engage with scientific explanations rather than personal testimony. Prior research has established that content style and audience composition shape user behavior on social platforms [40,68], and our results extend this principle to the specific domain of creator-led metabolic health content. This pattern is further supported by findings that channels with longer average comments had significantly higher positive-outcome rates (ρ=0.645; *P*=.03) and that false-negative rates increased substantially with comment length. These results indicate that the richer, more detailed forms of self-disclosure fostered by certain creator styles also generate more complex health narratives that automated extraction systems are less likely to capture fully. For researchers designing computational health discourse tools, this suggests that extraction frameworks must be calibrated not only to the health domain but also to the discourse norms of the specific communities being studied. For platform designers, these patterns suggest actionable interventions: features that surface testimonial density, link outcome claims to their evidentiary basis, or guide viewers toward content that matches their information needs could meaningfully reshape how patients encounter health testimony online [20]. The framework and ontology presented here provide the measurement infrastructure needed to evaluate such interventions at scale.

In the health natural language processing literature, classification systems have been optimized primarily for balanced performance, yielding precision levels insufficient for high-confidence corpus generation, in which every included observation must be valid [43,53]. Our precision-first rule-based architecture addresses this gap, exceeding the precision reported in comparable systems (Table 13) [43]. External validation confirms that this advantage extends beyond the development corpus [69]. The transformer baseline comparison, in which fine-tuned models achieved higher recall but lower precision, confirms that this advantage is architectural rather than data-dependent [42]. For the broader health natural language processing community, this finding supports a practical design principle: when the RO is corpus generation rather than individual case detection, rule-based architectures with domain-specific ontologies offer a precision advantage that current deep learning approaches do not match [43]. The construction and validation workflows are domain-agnostic and transferable to other chronic-disease communities and health-adjacent digital platforms, subject to ontology respecification and revalidation [60].

### Limitations and Future Directions

This study has limitations that define its scope and point to productive extensions. Commenters are a self-selected subset of viewers, and those experiencing positive outcomes may be more likely to comment, inflating the observed rate relative to the underlying population [29]. The sample selection strategy prioritized the 10 most-commented videos per channel, further biasing the sample toward viral content [68], and reply threads were excluded due to API constraints [70]. Manual validation was performed by a single domain-expert coder, with interrater reliability assessed through LLM-assisted annotation rather than independent human domain-expert validation [45,71]. The observed association between creator discourse style and testimonial characteristics is correlational and based on eleven channels, limiting causal inference and the generalizability of channel-level patterns. Additionally, the significantly higher false-negative rate among longer comments means that the framework may systematically under-capture outcomes in communities whose discourse norms encourage more detailed self-reporting, compounding the channel-level variation described above. Addressing these constraints through multicoder validation at scale and incorporating reply threads is a priority for future research.

The framework was developed and validated within a single dietary domain on a single platform. The ontology was engineered for TCR, and adapting it to other health domains requires modifying the ontology and revalidating it rather than direct transfer [60]. Results may not generalize to platforms with different demographics, moderation norms, or content formats [40,72]. While external validation on held-out channels confirmed precision transfer, 3 of 5 external channels yielded fewer than fifteen classifier-positive comments, precluding reliable per-channel estimates. Extending the framework to adjacent chronic-disease communities on YouTube and to other health-adjacent platforms represents a natural next step [61].

The nature of self-reported content imposes additional constraints. Outcomes cannot be independently verified, and users may misattribute improvements or conflate correlation with causation; the classification only confirms that users report these outcomes, not that the underlying health claims are accurate [73]. The framework extracts only positive outcomes, an asymmetry only partially mitigated by the supplementary ABSA analysis. Comments are point-in-time reports that preclude assessment of long-term sustainability [26]. Developing a dedicated negative-outcome extraction framework and expanding pattern libraries to close the recall gap are the most immediate methodological extensions, enabling a more complete characterization of health discourse in creator-led digital communities.

### Conclusions

This study establishes that creator-led metabolic-health YouTube content is a scalable, computationally viable source of self-reported health outcomes and presents a replicable, precision-first methodology for extracting and validating these outcomes at the corpus scale. Beyond the methodology, the breadth of reported outcomes, the influence of creator discourse style on the volume and nature of testimonial reporting, and the medication-discontinuation signal collectively position healthcasting as a phenomenon warranting sustained attention from the health informatics, health communication, and clinical research communities. As this attention grows, the precision-first architecture and hierarchical ontology provide a transferable methodological foundation for computational health discourse analysis across chronic-disease domains and digital platforms.

## Data Availability

The datasets generated or analyzed during this study are available in the GitHub repository. Additional data are included in this published article and its supplementary information files.

## Appendix

Multimedia Appendix 1 CREMLS reporting checklist.

Multimedia Appendix 2 Summary: videos and comments by channel.

Multimedia Appendix 3 Ontology structure with representative keyword and exclusion patterns.

Multimedia Appendix 4 Classification rule patterns.

Multimedia Appendix 5 External validation protocol and results.

Multimedia Appendix 6 Large language model–assisted annotation prompt.

Multimedia Appendix 7 Transformer baseline comparison.

Multimedia Appendix 8 Aspect-based sentiment analysis prompt design and few-shot examples.

Multimedia Appendix 9 Large language model annotation bias audit.

## Abbreviations

ABSA: aspect-based sentiment analysis
API: application programming interface
BERT: Bidirectional Encoder Representations from Transformers
CREMLS: Consolidated Reporting Guidelines for Prognostic and Diagnostic Machine Learning Modeling Studies
LLM: large language model
RO: research objective
RoBERTa: Robustly Optimized BERT Pretraining Approach
RQ: research question
TCR: therapeutic carbohydrate restriction
