# Supplementary material for: Self-Reported Health Outcomes in Metabolic Health YouTube Comments: Cross-Sectional Study and Rule-Based Natural Language Processing Framework Development and Validation
Source: J Med Internet Res. 2026 May 26;28:e94855. doi: 10.2196/94855 (PMC13250492; doi:10.2196/94855)
Supplement: Multimedia Appendix 1 [file jmir_v28i1e94855_app1.docx]

# Appendix 1: CREMLS Reporting Checklist

Completed checklist following the Consolidated Reporting Guidelines for Prognostic and Diagnostic Machine Learning Modeling Studies (CREMLS; Klement and El Emam, 2023). Items are mapped to manuscript sections where each reporting requirement is addressed. N/A indicates items not applicable to the study design (rule-based text classification of public social media data).

| **Item** | **CREMLS Reporting Item** | **Reported?** | **Manuscript Section** | **Details/Comments** |
| --- | --- | --- | --- | --- |
| **Category 1: Study Details** |  |  |  |  |
| 1.1 | Medical/clinical task of interest | **Yes** | Introduction, Research Gap | Classification of self-reported positive health outcomes from YouTube comments on metabolic health content |
| 1.2 | Research question | **Yes** | Introduction, Research Questions | RQ1-RQ4 explicitly stated with prevalence, distribution, channel variation, and classification accuracy objectives |
| 1.3 | Current medical/clinical practice | **Yes** | Background; Health Information Extraction From Social Media; Text Classification Approaches | Current social media health mining, pharmacovigilance, and text classification approaches reviewed |
| 1.4 | Known predictors and confounders | **Yes** | Limitations, Internal Validity; Limitations, Construct Validity | Selection bias, single-coder validation, positive reporting bias, self-report accuracy, kappa paradox discussed |
| 1.5 | Overall study design | **Yes** | Methods, Research Design Overview; Abstract | Observational, cross-sectional computational study; three construction phases with integrated validation studies and supplementary contextualization analysis |
| 1.6 | Medical institutional settings | **N/A** | Methods, Data Collection | Public YouTube data via API; no institutional clinical setting. Platform and channel selection criteria described |
| 1.7 | Target population | **Yes** | Methods, Data Collection; Introduction, Background and Motivation | YouTube commenters on 11 TCR-focused Healthcasting channels (N=43,111 comments, 37,458 unique authors) |
| 1.8 | Intended use of model | **Yes** | Methods, Research Design Overview; Discussion, Conclusions | High-confidence corpus generation for downstream health outcomes research; not clinical decision support |
| 1.9 | Existing model performance benchmarks | **Yes** | Introduction, Research Gap; Results, Table 13; Discussion, Comparison With Prior Work | Prior systems reported 80-90% precision; seven studies compared in literature comparison table |
| 1.10 | Ethical and regulatory approvals | **Yes** | Methods, Ethical Considerations and Data Governance | All five JMIR-required points: ethics review rationale, informed consent waiver, privacy protections, compensation, no identifiable images |
| **Category 2: Data Description** |  |  |  |  |
| 2.1 | Inclusion and exclusion criteria | **Yes** | Methods, Data Collection | Channel selection criteria (credentials, subscribers, focus, active comments); video selection (10 most-commented); API deduplication |
| 2.2 | Methods of data collection | **Yes** | Methods, Data Collection | YouTube Data API v3; 10 most-commented videos per channel; up to 2,000 comments per video; collection date 2 January 2026 |
| 2.3 | Bias from data collection method | **Yes** | Limitations, Internal Validity | Selection bias, viral content bias, positive reporting bias, reply thread exclusion discussed |
| 2.4 | Data characteristics | **Yes** | Methods, Data Collection; Results, Table 1; Methods, Phase 1 | N=43,111 comments; 37,458 authors; Nov 2013-Jan 2026; length distribution; channel-level statistics in Table 1 |
| 2.5 | Data transformations and preprocessing | **Yes** | Methods, Phase 2 Ontology Development; Methods, Phase 3 Classification Framework | Keyword matching with whole-word regex; three-stage pipeline; exclusion filtering; case-insensitive matching |
| 2.6 | Known quality issues | **Yes** | Methods, Data Collection; Limitations | API pagination duplicates; self-report accuracy; comment deletion/moderation; temporal instability |
| 2.7 | Sample size calculation | **Yes** | Methods, Validation Studies | Precision: n=500, 95% CI, 4% margin of error. Recall: n=510, disproportionate stratification by comment length |
| 2.8 | Data availability | **Yes** | Discussion, Conclusions | GitHub repository with ontology, code, validation protocols. Raw comments restricted per YouTube API Terms of Service |
| **Category 3: Modeling Methodology** |  |  |  |  |
| 3.1 | Strategies for handling missing data | **N/A** | - | Text classification of complete comment units; no missing feature values |
| 3.2 | Strategies for addressing class imbalance | **Yes** | Methods, Research Design Overview; Methods, Validation Studies | 4.15% positive prevalence; precision-optimized design addresses imbalance; disproportionate stratified sampling for recall |
| 3.3 | Strategies for reducing dimensionality | **Yes** | Methods, Phase 2 Ontology Development | 35-aspect hierarchical ontology with 520 keywords; iterative corpus-driven refinement; exclusion patterns for disambiguation |
| 3.4 | Strategies for handling outliers | **Yes** | Methods, Phase 3 Classification Framework | Three-stage exclusion filtering removes questions, third-party reports, hypothetical language, negated outcomes, general advice |
| 3.5 | Strategies for data augmentation | **N/A** | - | Rule-based system; no training data augmentation used |
| 3.6 | Strategies for model pretraining | **Partial** | Multimedia Appendix 6 | N/A for primary rule-based model. Transformer baselines used standard pretrained weights with fine-tuning |
| 3.7 | Rationale for selecting algorithm | **Yes** | Methods, Research Design Overview; Discussion, Comparison With Prior Work | Rule-based for precision control, interpretability, deterministic reproducibility, zero labeled training data requirement |
| 3.8 | Method of evaluating performance during training | **Yes** | Methods, Phase 2 Ontology Development | Two validation rounds (coverage testing, precision refinement); 20 random samples per aspect; ontology locked before Phase 3 |
| 3.9 | Method for hyperparameter tuning | **Partial** | Methods, Phase 3; Multimedia Appendix 6 | Rule-based: iterative keyword/pattern refinement. Transformers: stratified 5-fold CV with standard hyperparameters |
| 3.10 | Model output adjustments | **Yes** | Methods, Phase 3 Classification Framework | Three-stage pipeline with conservative exclusion thresholds; deterministic binary output, no post-hoc calibration |
| **Category 4: Model Evaluation** |  |  |  |  |
| 4.1 | Performance metrics | **Yes** | Results, Classification Performance; Tables 3-4 | Precision, recall, Wilson score 95% CIs; error analysis by type; channel-level and length-strata variation |
| 4.2 | Cost or consequence of errors | **Yes** | Methods, Research Design Overview; Discussion, Principal Findings | False positives contaminate corpus; false negatives reduce power without introducing bias. Trade-off explicitly justified |
| 4.3 | Results of internal validation | **Yes** | Results, Classification Performance; Results, Inter-Rater Reliability; Tables 3-5 | Precision 97.6% (488/500); recall 56.2%; Cohen kappa across 3 pairs and 4 dimensions; error analysis |
| 4.4 | Final model hyperparameters | **Yes** | Methods, Phase 3; Appendices A-B | Complete ontology (35 aspects, 520 keywords), outcome indicator patterns, exclusion filters in appendices and GitHub |
| 4.5 | External validation | **Yes** | Results, Classification Performance; Multimedia Appendix 4 | 12,653 comments from 5 unseen channels; 93.4% precision (227/243); overlapping CIs with development corpus |
| 4.6 | Data shift and drift characteristics | **Yes** | Limitations, External Validity; Limitations, Reliability | Temporal span Nov 2013-Jan 2026; platform changes; domain and platform specificity discussed |
| **Category 5: Model Explainability** |  |  |  |  |
| 5.1 | Important features and relation to outcomes | **Yes** | Results, Health Aspect Analysis; Results, Error Analysis; Table 7 | Top 10 aspects by frequency; false negative analysis by aspect; structural pattern mismatch as dominant miss mechanism |
| 5.2 | Plausibility of model outputs | **Yes** | Discussion, Comparison With Prior Work; Results, Sentiment Contextualization | Outcomes map to clinically recognized TCR effects; supplementary ABSA broadly consistent with positive predominance (indicative, not confirmatory); negative aspects align with known adaptation |
| 5.3 | Interpretation by end user | **Yes** | Discussion, Conclusions | Intended users: health informatics researchers, public health surveillance, platform designers, health communication researchers |

Reference: Klement W, El Emam K. Consolidated Reporting Guidelines for Prognostic and Diagnostic Machine Learning Modeling Studies: Development and Validation. J Med Internet Res. 2023;25:e48763. doi:10.2196/48763
