# Supplementary material for: Self-Reported Health Outcomes in Metabolic Health YouTube Comments: Cross-Sectional Study and Rule-Based Natural Language Processing Framework Development and Validation
Source: J Med Internet Res. 2026 May 26;28:e94855. doi: 10.2196/94855 (PMC13250492; doi:10.2196/94855)
Supplement: Multimedia Appendix 3 [file jmir_v28i1e94855_app3.docx]

# Appendix 3: Ontology Structure with Representative Keyword and Exclusion Patterns

Table A1 presents five representative aspects from the 35-aspect ontology, illustrating the keyword matching and exclusion pattern structure across all three research objectives. The complete ontology specification, including all keywords and exclusions for all 35 aspects, is available in the supplementary materials repository (the Reproducibility Statement).

**Table A1.** *Representative ontology aspects with keyword and exclusion patterns. Full ontology (35 aspects, 520 total keywords) available in supplementary materials.*

| **Aspect** | **RO** | **Keywords (representative)** | **Exclusions** | **n** |
| --- | --- | --- | --- | --- |
| RO1.3: Psychological Well-Being | RO1 | anxiety, anxious, depression, depressed, mood, stress, mental health, happier, calm, peaceful, irritable, well-being, … | adhd, add, attention deficit | 22 |
| RO1.6: Pain & Inflammation | RO1 | pain, ache, inflammation, joint pain, back pain, chronic pain, stiffness, swelling, headache, migraine, soreness, … | arthritis, fibromyalgia, gout, rheumatoid | 24 |
| RO2.1: Anthropometric Changes | RO2 | weight, lost weight, weight loss, pounds, lbs, kg, waist, inches, bmi, body fat, fat loss, dress size, pant size, visceral fat, … | (none) | 28 |
| RO2.2: Glycemic Control | RO2 | glucose, blood sugar, a1c, hba1c, fasting glucose, fasting insulin, insulin resistance, cgm, glucometer, sugar level, … | diabetes, diabetic, type 2, t2d, prediabetes | 21 |
| RO3.1: Type 2 Diabetes | RO3 | diabetes, diabetic, type 2 diabetes, t2d, prediabetes, prediabetic, reversed diabetes, metformin, type two diabetes, … | (none) | 15 |

Note: Keywords are matched case-insensitively. Exclusions redirect matches to more specific disease aspects (eg, “arthritis” in a pain context is classified under RO3.17 rather than RO1.6). The column n indicates total keyword count per aspect.

**RO1 - Subjective Well-Being (9 aspects):** RO1.1 Cognitive Function, RO1.2 Energy & Vitality, RO1.3 Psychological Well-Being, RO1.4 Sleep Quality, RO1.5 Appetite & Satiety, RO1.6 Pain & Inflammation, RO1.7 Digestive Health, RO1.8 Skin Health, RO1.9 Hormonal & Menstrual Health

**RO2 - Tool-Mediated Validation (8 aspects):** RO2.1 Anthropometric Changes, RO2.2 Glycemic Control, RO2.3 Blood Pressure, RO2.4 Lipid Profile, RO2.5 Inflammatory Markers, RO2.6 Liver Function, RO2.7 Kidney Function, RO2.8 Hormonal Markers

**RO3 - Disease Specificity (18 aspects):** RO3.1 Type 2 Diabetes, RO3.2 Fatty Liver Disease, RO3.3 Cardiovascular Disease, RO3.4 Hypertension, RO3.5 PCOS, RO3.6 Neurodegenerative Disease, RO3.7 Chronic Kidney Disease, RO3.8 Gout, RO3.9 Cancer, RO3.10 Osteoporosis, RO3.11 Stroke, RO3.12 ADHD, RO3.13 Thyroid Disease, RO3.14 Inflammatory Bowel Disease, RO3.15 Autoimmune Disease, RO3.16 Fibromyalgia & Neuropathy, RO3.17 Arthritis, RO3.18 Gallbladder Disease
