# Supplementary material for: Self-Reported Health Outcomes in Metabolic Health YouTube Comments: Cross-Sectional Study and Rule-Based Natural Language Processing Framework Development and Validation
Source: J Med Internet Res. 2026 May 26;28:e94855. doi: 10.2196/94855 (PMC13250492; doi:10.2196/94855)
Supplement: Multimedia Appendix 4 [file jmir_v28i1e94855_app4.docx]

# Appendix 4: Classification Rule Patterns

The three-stage classification pipeline (the Classification Framework section) relies on outcome indicator patterns (Stage 2) and exclusion filter patterns (Stage 3). Tables B1 and B2 present the pattern categories with representative regular expressions.

**Table B1.** *Outcome indicator categories with representative patterns (Stage 2). Complete pattern set (110 patterns) available in supplementary materials.*

| **Category** | **n** | **Representative Patterns** |
| --- | --- | --- |
| Quantified Change | 20 | lost\s+\d+\s*(pound\|lb\|kg) ; down\s+\d+\s*(pound\|lb\|kg) ; a1c\s*(dropped\|went\s+down\|is\s+now)\s+\d |
| Symptom Cessation | 32 | (pain\|ache)\s*(is\|was)?\s*(gone\|disappeared) ; (brain\s+fog)\s*(is\|was)?\s*(gone\|lifted\|cleared) |
| Reversal/Remission | 16 | reversed\s+(my\|the)?\s*(type\s*2\s*)?(diabetes) ; no\s+longer\s+(have\|need)\s+(diabetes\|insulin) |
| Medication Discontinuation | 15 | off\s+(my\s+)?(metformin\|insulin\|statins?\|medication) ; stopped\s+(taking\s+)?(metformin\|insulin) |
| Explicit Improvement | 18 | my\s+(a1c\|blood\s+sugar)\s*(has\s+)?(improved\|normalized) ; my\s+(energy\|sleep\|mood)\s*(has\s+)?(improved) |
| Temporal Improvement | 9 | since\s+(starting\|going)\s+(keto\|carnivore).{0,50}(lost\|improved) ; after\s+\d+\s*(week\|month)s?.{0,30}(lost\|improved) |

**Table B2.** *Exclusion filter categories with representative patterns (Stage 3). Complete pattern set (45 patterns) available in supplementary materials.*

| **Category** | **n** | **Representative Patterns** |
| --- | --- | --- |
| Questions | 6 | \?\s*$ ; ^(can\|does\|will\|should)\s+(this\|keto\|carnivore) |
| Negation | 12 | (didn't\|haven't)\s+(lose\|help\|work\|improve) ; (no\|not\s+any)\s+(change\|improvement\|results) |
| Third-Party References | 5 | ^my\s+(husband\|wife\|mom\|dad\|friend\|son\|daughter) ; ^(he\|she\|they)\s+(lost\|reversed\|improved) |
| Future Intent | 9 | ^i\s+(want\|need\|hope\|plan)\s+to\s+(lose\|reverse\|improve) ; ^(trying\|hoping)\s+to\s+(lose\|reverse) |
| General Statements | 7 | ^(keto\|carnivore\|fasting)\s+(helps?\|can\|will) ; ^(studies\|research)\s+(show\|prove) |
| Engagement Only | 6 | ^(great\|good\|amazing)\s+(video\|content\|info) ; ^(thanks?\|thank\s+you) |

Note: Patterns are applied sequentially; a comment matching any exclusion pattern is removed from the positive pool regardless of outcome indicator matches. Regular expressions use case-insensitive matching.
