# Supplementary material for: Self-Reported Health Outcomes in Metabolic Health YouTube Comments: Cross-Sectional Study and Rule-Based Natural Language Processing Framework Development and Validation
Source: J Med Internet Res. 2026 May 26;28:e94855. doi: 10.2196/94855 (PMC13250492; doi:10.2196/94855)
Supplement: Multimedia Appendix 5 [file jmir_v28i1e94855_app5.docx]

# Appendix 5: External Validation Protocol and Results

***External Validation Protocol***

To assess generalisability beyond the development corpus, external validation was conducted on 12,653 comments collected from five YouTube channels not included in the original 11-channel sample. Channels were selected by an independent co-author (A.Z.) based on topical relevance to ketogenic, carnivore, and metabolic health dietary content, with zero overlap with the development channels. The five channels were: Georgia Ede MD (n=88 comments), Robert Kiltz MD (n=187), Sten Ekberg DC (n=10,838), Chris Palmer MD (n=548), and Ted Naiman MD (n=992). For each channel, comments were collected from the 10 most-commented videos using the YouTube Data API v3, following the same protocol as the development corpus.

The classifier was applied to the external corpus without modification. All 243 comments classified as positive health outcomes were exhaustively verified by the first author (R.R.) through manual coding to assess precision. This census approach was adopted because the smaller number of classifier-positive comments in the external corpus permitted exhaustive verification, which is methodologically stronger than subsampling. For recall estimation, a stratified random sample of 487 comments classified as non-positive was drawn (100 per channel, or all available where fewer than 100 existed; random seed = 42) and manually coded to identify false negatives. Precision is reported as an exact figure; recall is estimated by extrapolating per-channel false negative rates to the full negative population, with 95% Wilson score confidence intervals.

## External Validation

The classifier achieved a precision of 93.4% (227/243; 95% CI 89.6%-95.9%) on the external corpus, compared with 97.6% (95% CI 95.8%-98.7%) on the development corpus. The confidence intervals overlap, indicating that the classifier maintains high precision on genuinely unseen content. Of the 16 false positives, the most common error patterns were general health mentions without personal outcomes (n=7), third-party reports (n=5), and statements of intent without reported results (n=4).

Recall was estimated at 50.1% (95% CI 31.4%-59.1%), comparable to the estimate for the development corpus of 56.2%. Eleven false negatives were identified in the 487-comment recall sample (2.3% false negative rate). False negative rates varied across channels: Georgia Ede MD exhibited an elevated rate (8/87, 9.2%; 95% CI 4.7%-17.1%), while the remaining channels showed rates of 0.0%-2.0%. The Georgia Ede channel’s higher rate appeared attributable to mental health recovery narratives involving psychiatric medication changes, a content pattern at the boundary of the current ontology’s metabolic health scope. One false negative was in Spanish, confirming that the English-language ontology does not capture non-English testimonials.

The lower positive outcome rate in the external corpus (1.92% vs. 9.64%) reflects the broader content mix of the validation channels rather than classifier degradation: high-subscriber channels with viral informational content attract comments that use health vocabulary without reporting personal outcomes. The classifier correctly discriminated between health-related commentary and genuine personal health testimonials.
