# Supplementary material for: Self-Reported Health Outcomes in Metabolic Health YouTube Comments: Cross-Sectional Study and Rule-Based Natural Language Processing Framework Development and Validation
Source: J Med Internet Res. 2026 May 26;28:e94855. doi: 10.2196/94855 (PMC13250492; doi:10.2196/94855)
Supplement: Multimedia Appendix 6 [file jmir_v28i1e94855_app6.docx]

# Appendix 7: LLM-Assisted Annotation Prompt

Both GPT-4o and GPT-4.1 received the identical structured prompt below (temperature = 0.0, with 10 comments per batch). The prompt comprised four sections: task definition with coding guidelines, the 35-aspect ontology reference, 28 few-shot exemplars with ground-truth codings, and the comment batch to be coded. The complete prompt (387 lines) is available in the supplementary materials repository (the Reproducibility Statement). The task definition and representative exemplars are reproduced here.

**Task Definition (System Message):**

*"You are an independent annotation coder for a health informatics research study. Your task is to evaluate YouTube comments that were automatically classified as containing positive health outcomes related to dietary interventions (keto, carnivore, low-carb, intermittent fasting). You must independently code each comment on FOUR dimensions: (1) is_positive_outcome (Yes / No / Unclear), (2) is_personal (Yes / No), (3) is_definite (Yes / No), (4) aspect_correct (Yes / No / Partial)."*

**Key Coding Guidelines (excerpt):**

*"Code INDEPENDENTLY based on the comment text. Do not assume the automated system is correct. A comment can be personal AND positive but NOT definite. Mixed outcomes (some positive, some negative) should generally be coded as Unclear unless the positive outcome is clearly dominant. Comments that describe negative outcomes from the diet are No."*

**Few-Shot Exemplars (28 total, 6 categories):**

Exemplars were drawn from the 500-sample validation set and grouped into six categories to ensure coverage of boundary cases. Table C1 summarizes the exemplar distribution and shows one representative from each category.

**Table C1.** *Few-shot exemplar categories with representative examples. A full set of 28 exemplars is available in the supplementary materials.*

| **Category** | **n** | **Representative Exemplar (abbreviated)** | **Coding** |
| --- | --- | --- | --- |
| Clear true positive | 10 | "4 months into keto…got off a ton of meds and all my diseases were gone…lost 155 pounds" | Yes / Yes / Yes / Yes |
| Clear negative (FP) | 5 | "My son has been on carnivore…lost 285 lbs…but now he is not [well], no energy, can’t work…" | No / No / No / Yes |
| Unclear/ambiguous | 5 | "I did lose weight, I lost 30 lbs…I also had a heart attack at the end of that year" | Unclear / Yes / Yes / Partial |
| Positive, not personal | 3 | "My girlfriend reversed her type 2 diabetes with Keto…off medication for over 20 years" | Yes / No / Yes / Yes |
| Positive, not definite | 2 | "Been doing this 2 days and lost 2 pounds already!…I’ve got 62 more pounds to lose" | Yes / Yes / No / Yes |
| Aspect assignment issue | 3 | "Healed my FATTY LIVER DISEASE, PRE-DIABETES…ZERO pre-cancerous polyps" [auto-assigned RO3.9 incorrectly] | Yes / Yes / Yes / Partial |

Note: Coding columns show is_positive_outcome / is_personal / is_definite / aspect_correct. Comment texts are abbreviated; full texts are available in supplementary materials. Models were instructed to respond only with a JSON array containing all four coding dimensions, along with optional notes.
