# Supplementary material for: Self-Reported Health Outcomes in Metabolic Health YouTube Comments: Cross-Sectional Study and Rule-Based Natural Language Processing Framework Development and Validation
Source: J Med Internet Res. 2026 May 26;28:e94855. doi: 10.2196/94855 (PMC13250492; doi:10.2196/94855)
Supplement: Multimedia Appendix 7 [file jmir_v28i1e94855_app7.docx]

# Appendix 6: Transformer Baseline Comparison

## Transformer Baseline Comparison

To contextualize the rule-based framework’s performance, we trained BERT-base-uncased and RoBERTa-base classifiers on the combined precision-validation and recall-expansion datasets (n=836 unique comments; 347 positive, 489 negative) using stratified five-fold cross-validation (see Multimedia Appendix 6 for full experimental details). Table F1 summarizes the comparison across key metrics.

**Table F1.** *Summary comparison of rule-based framework versus transformer baselines (BERT-base-uncased, RoBERTa-base) for classifying self-reported health outcomes in English-language YouTube comments on 11 metabolic health channels (N=43,111 unique comments, November 2013 to January 2026). Transformers trained on n=836 labeled comments. CV metrics report mean and SD across five stratified folds. Precision subset: evaluation on the 326 precision-validation comments. FN recovered: false negatives from the recall-expansion sample (n=510) detected by each model.*

| **Metric** | **Rule-Based Framework** | **BERT-base** | **RoBERTa-base** |
| --- | --- | --- | --- |
| CV Precision | 97.6% | 87.0 (SD 5.3)% | 88.2 (SD 3.9)% |
| CV Recall | 56.2% | 93.4 (SD 3.3)% | 95.7 (SD 0.9)% |
| CV F1-score | 71.4% | 89.9 (SD 3.2)% | 91.8 (SD 2.3)% |
| CV ROC-AUC | N/A | 95.9 (SD 1.7)% | 96.5 (SD 2.1)% |
| Precision subset (n=326) | 97.6% | 99.7% | 99.0% |
| FN recovered (of 27) | 0/27 (0%) | 22/27 (81.5%) | 24/27 (88.9%) |
| Training data required | None | Yes (n=836) | Yes (n=836) |
| Interpretability | Full (rule trace) | Low (black box) | Low (black box) |
| Reproducibility | Deterministic | Stochastic | Stochastic |

Both transformer models achieved substantially higher F1-scores than the rule-based framework (+18.5 to +20.4 percentage points), driven primarily by their superior recall (93.4% and 95.7% versus 56.2%). However, the rule-based framework maintained the highest precision (97.6%), exceeding both BERT (87.0%) and RoBERTa (88.2%) by approximately 10 percentage points. On the precision-validation subset (n=326), both transformers achieved precision comparable to or exceeding the rule-based framework (BERT: 99.7%; RoBERTa: 99.0%), confirming that the framework’s positive classifications are robust. The recall-expansion subset analysis showed that RoBERTa recovered 24 of 27 false negatives (88.9%) and BERT recovered 22 of 27 (81.5%), though with additional false positives (42 and 49, respectively) on the heavily skewed negative subset (483 of 510 comments).
