# Supplementary material for: Self-Reported Health Outcomes in Metabolic Health YouTube Comments: Cross-Sectional Study and Rule-Based Natural Language Processing Framework Development and Validation
Source: J Med Internet Res. 2026 May 26;28:e94855. doi: 10.2196/94855 (PMC13250492; doi:10.2196/94855)
Supplement: Multimedia Appendix 8 [file jmir_v28i1e94855_app8.docx]

# Appendix 8: ABSA Prompt Design and Few-Shot Examples

The Aspect-Based Sentiment Analysis (the Sentiment Contextualization section) employed the following prompt design. Both GPT-4o and GPT-4.1 received identical instructions (temperature = 0.0, batches of 10 comments). The prompt comprised: (1) a system-level task definition specifying the 17 health aspect categories and sentiment classification rules, and (2) seven few-shot exemplars demonstrating the expected JSON output format across the four sentiment categories (positive, negative, neutral, mixed) plus non-health-related comments. The complete system prompt and all few-shot examples are reproduced below.

**D.1 System Prompt (Task Definition)**

You are an expert health informatics coder performing Aspect-Based Sentiment Analysis (ABSA) on YouTube comments from metabolic health channels.

These channels discuss Therapeutic Carbohydrate Restriction (TCR), including ketogenic, low-carb, carnivore, and intermittent fasting approaches.

For each comment, determine: (1) health_related: Is this comment related to health? (true/false); (2) health_aspects: If health_related is true, list ALL health aspects mentioned with aspect category, sentiment (positive/negative/neutral/mixed), and confidence (high/medium/low); (3) overall_health_sentiment: The overall health sentiment of the comment.

**Health Aspect Categories (17):**

weight_change (weight loss/gain, body composition); blood_sugar (HbA1c, glucose, insulin, diabetes management); energy_mood (energy levels, mood, mental clarity, brain fog); pain_inflammation (joint pain, chronic pain, inflammation); cardiovascular (blood pressure, cholesterol, heart health); digestive (IBS, gut health, bloating, acid reflux); skin (acne, eczema, psoriasis); sleep (sleep quality, insomnia); medication (starting/stopping/changing medication); autoimmune (autoimmune conditions, RA, lupus, MS); mental_health (anxiety, depression, ADHD); cancer (any cancer-related mentions); hormonal (thyroid, PCOS, fertility, menopause); neurological (epilepsy, seizures, neuropathy, Alzheimer’s); general_wellbeing (general health improvement, vitality); diet_adherence (difficulty/ease of following diet, cravings, sustainability); other_health (any health aspect not covered above).

**Coding Rules:**

Code what the comment ACTUALLY says, not what you infer. Third-person reports (“my mom lost weight”) count. Questions about health (“will keto help my diabetes?”) are neutral, not positive. Gratitude TO the creator (“thank you doctor”) is NOT health-related unless it mentions a specific health outcome. “I feel great” in a health context = positive general_wellbeing. If unsure about sentiment, use “neutral”.

**D.2 Few-Shot Examples (7 Exemplars)**

The following seven exemplars were provided to both models as in-context demonstrations, covering positive, negative, neutral, mixed, and non-health-related comment categories.

**Example 1 (Positive, multi-aspect):**

Comment: “I lost 30 pounds in 3 months on keto and my A1C went from 9.1 to 5.4! My doctor was shocked.”

→ {health_related: true, health_aspects: [{aspect: “weight_change”, sentiment: “positive”}, {aspect: “blood_sugar”, sentiment: “positive”}], overall_health_sentiment: “positive”}

**Example 2 (Non-health-related):**

Comment: “Great video as always Dr. Berg! Keep up the good work!”

→ {health_related: false, health_aspects: [], overall_health_sentiment: null}

**Example 3 (Negative):**

Comment: “I tried carnivore for 6 weeks and my LDL went through the roof. My doctor is very concerned.”

→ {health_related: true, health_aspects: [{aspect: “cardiovascular”, sentiment: “negative”}], overall_health_sentiment: “negative”}

**Example 4 (Neutral):**

Comment: “I have type 2 diabetes and I’m thinking about trying this approach. Has anyone had experience?”

→ {health_related: true, health_aspects: [{aspect: “blood_sugar”, sentiment: “neutral”}], overall_health_sentiment: “neutral”}

**Example 5 (Mixed):**

Comment: “Lost a lot of weight which is great but my sleep has gotten worse since starting keto”

→ {health_related: true, health_aspects: [{aspect: “weight_change”, sentiment: “positive”}, {aspect: “sleep”, sentiment: “negative”}], overall_health_sentiment: “mixed”}

**Example 6 (Non-health-related):**

Comment: “This is just big pharma propaganda. Wake up people!”

→ {health_related: false, health_aspects: [], overall_health_sentiment: null}

**Example 7 (Negative):**

Comment: “My HbA1c worsened after going back to carbs”

→ {health_related: true, health_aspects: [{aspect: “blood_sugar”, sentiment: “negative”}], overall_health_sentiment: “negative”}

Note. The seven exemplars were designed to cover: multi-aspect positive (Example 1), single-aspect negative (Examples 3 and 7), neutral/questioning (Example 4), mixed sentiment with competing aspects (Example 5), and non-health-related comments including both creator appreciation and political commentary (Examples 2 and 6). Confidence ratings (high/medium/low) were included in all exemplar outputs but omitted here for brevity.
