# Supplementary material for: Self-Reported Health Outcomes in Metabolic Health YouTube Comments: Cross-Sectional Study and Rule-Based Natural Language Processing Framework Development and Validation
Source: J Med Internet Res. 2026 May 26;28:e94855. doi: 10.2196/94855 (PMC13250492; doi:10.2196/94855)
Supplement: Multimedia Appendix 9 [file jmir_v28i1e94855_app9.docx]

# Appendix 9: LLM Annotation Bias Audit

To assess systematic measurement bias in the LLM-assisted inter-rater reliability assessment (Reviewer S, Comment S2), full confusion matrices were computed between each coder pair (Human, GPT-4o, GPT-4.1) across four coding dimensions: positive health outcome, first-person testimony, definite outcome, and aspect assignment accuracy. McNemar tests assessed directional disagreement; chi-square tests of homogeneity assessed overall label distribution shifts. All analyses used the matched subset of n=472 comments coded by all three coders.

**Table S1. Summary of LLM annotation bias across coding dimensions (n=472).**

| **Dimension** | **Comparison** | **Agreement** | **McNemar P** | **Chi-square P** | **Direction of Bias** |
| --- | --- | --- | --- | --- | --- |
| Positive Outcome | Human vs GPT-4o | 83.7% | *P*<.001 | *P*<.001 | GPT-4o classifies 30 fewer as positive |
| Positive Outcome | Human vs GPT-4.1 | 87.0% | *P*<.001 | *P*<.001 | GPT-4.1 shifts 9 from positive |
| First-Person | Human vs GPT-4o | 98.1% | *P*=.004 | NS (*P*=.118) | Near-perfect; minor bias (9 cases) |
| First-Person | Human vs GPT-4.1 | 99.4% | NS (*P*=.250) | NS (*P*=.643) | No significant bias |
| **Definiteness** | **Human vs GPT-4o** | **66.9%** | ***P*<.001** | ***P*<.001** | **GPT-4o downgrades 135 from definite** |
| **Definiteness** | **Human vs GPT-4.1** | **82.4%** | ***P*=.048** | NS (*P*=.078) | **GPT-4.1 downgrades 51 (net 19)** |
| Aspect Accuracy | Human vs GPT-4o | 61.2% | *P*<.001 | *P*<.001 | GPT-4o shifts 154 from correct to partial/incorrect |
| Aspect Accuracy | Human vs GPT-4.1 | 56.6% | *P*<.001 | *P*<.001 | GPT-4.1 shifts 176 from correct to partial/incorrect |

Note: Bold rows indicate dimensions with the strongest bias signal. McNemar P tests directional disagreement between coder pairs on each class. Chi-square P tests overall label distribution homogeneity. NS = not significant at alpha = .05. Definiteness shows the largest bias: GPT-4o downgrades 135/472 (28.6%) comments from definite to indefinite relative to the human coder; GPT-4.1 shows a smaller but still significant shift (51 downgrades, net 19). First-person testimony is the most reliable dimension (98-99% agreement). Full confusion matrices and per-class McNemar tests are available in the supplementary materials repository.

Both LLMs exhibited systematic negative bias (stricter classification than the human coder), confirming the characterization of measurement bias raised by Reviewer S. GPT-4o demonstrated stronger definiteness bias than GPT-4.1 (135 vs 51 downgrades from definite to indefinite). First-person testimony was the most reliable dimension, with near-perfect agreement (98.1% and 99.4%). The cross-model comparison (GPT-4o vs GPT-4.1) yielded 94.3% agreement on the primary dimension with raw kappa = 0.771, providing convergent evidence that the coding task is well-specified despite individual model-level biases. These findings indicate that LLM-based inter-rater reliability estimates in this study are conservative: disagreements predominantly reflect LLM strictness rather than ambiguity in the coding scheme.
